# Transfer hydrogenations catalyzed by streptavidin-hosted secondary amine organocatalysts†

**DOI:** 10.1039/d0cc08142f

**Published:** 2021-01-14

**Authors:** Nicolò Santi, Louis C. Morrill, Katarzyna Świderek, Vicent Moliner, Louis Y. P. Luk

**Affiliations:** School of Chemistry, Main Building, Cardiff University Cardiff CF10 3AT UK lukly@cardiff.ac.uk +44 (0)29 2251 0161; Cardiff Catalysis Institute, School of Chemistry, Main Building, Cardiff University Cardiff CF10 3AT UK; Departament de Química Física i Analítica, Universitat Jaume I Castelló 12071 Spain

## Abstract

Here, the streptavidin–biotin technology was applied to enable organocatalytic transfer hydrogenation. By introducing a biotin-tethered pyrrolidine (1) to the tetrameric streptavidin (**T-Sav**), the resulting hybrid catalyst was able to mediate hydride transfer from dihydro-benzylnicotinamide (BNAH) to α,β-unsaturated aldehydes. Hydrogenation of cinnamaldehyde and some of its aryl-substituted analogues was found to be nearly quantitative. Kinetic measurements revealed that the T-Sav:1 assembly possesses enzyme-like behavior, whereas isotope effect analysis, performed by QM/MM simulations, illustrated that the step of hydride transfer is at least partially rate-limiting. These results have proven the concept that **T-Sav** can be used to host secondary amine-catalyzed transfer hydrogenations.

## Abstract 2

Streptavidin-based secondary amine enables organocatalytic hydride reduction.

## Graphical abstract

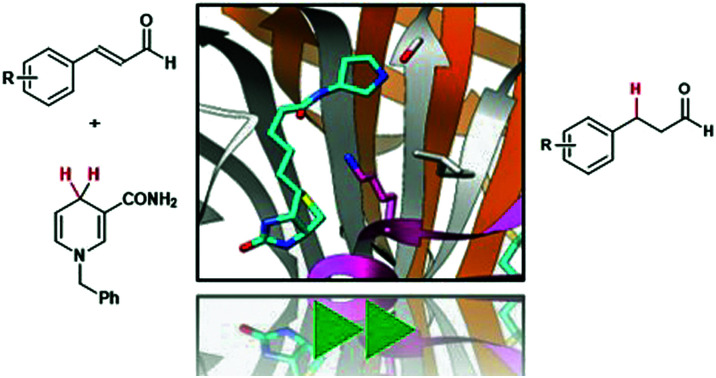

In biological systems, nicotinamide is frequently used in the transfer of electrons and hydrogen atoms^1,2^ and is typically found in the form of NAD(P)H attached to an adenosine dinucleotide appendix.^3^ While the adenosine dinucleotide motif is crucial for molecular recognition and metabolic regulation in cells,^4^ this appendix is a large and complex molecular burden that needs to be addressed when it comes to synthetic applications.^5^ Consequently, various cofactor recycling systems have been designed.^6,7^ In an alternative and potentially simpler approach, small organic molecules can be used as hydride donors,^8,9^ such as the Hantzsch ester,^10^ dihydro-benzylnicotinamide (BNAH) and their derivatives.^11,12^ Nevertheless, there are only a handful of protein-based catalysts reported to use simple hydride donors for reactions, including ene-reductases (ERs),^6,13^ salicylate (SalH), *para*-hydroxybenzoate (PHBH) and hydroxybenzoate (3HB6H) hydroxylases,^14^ cytochrome P450,^15^ 2-hydroxybiphenyl 3-monooxygenase (HbpA),^16^ Old Yellow Enzyme (TsOYE),^17^ and glucose dehydrogenases (GDH).^18^ Here, we aim to expand this collection by proving the concept that organocatalytic artificial enzymes can catalyze transfer hydrogenation using BNAH as a hydride source.^19^

Artificial enzymes can be created by docking chemical catalysts into a designated protein scaffold, and the resulting complex can potentially use hydride donors for reactions.^7^ Different approaches such as computational design, genetic code expansion, supramolecular approaches and amino acid modification have been used to accommodate chemical catalytic systems for bioorthogonal reactions.^19–25^ Previously, the streptavidin–biotin technology has been applied to host Ru and Ir-mediated transfer hydrogenation with the use of NAD(P)H and formic acid as hydride donors.^7,26–28^ Recently, streptavidin has also been used to host organocatalysis, including secondary amine, anion–π and DMAP catalysis;^19,20,23,29,30^ however, the reaction profile of these organocatalytic systems remains largely unexplored, and their possibility of mediating organocatalytic transfer hydrogenation reaction has not been tested.

Here, we demonstrate that secondary amine hosted by streptavidin can be used to catalyze transfer hydrogenation reactions (Fig. 1). Hybrid catalysts have been created by introducing biotinylated secondary amines (**1** and **2**) to the tetrameric streptavidin (**T-Sav**) or the monomeric counterpart (**M-Sav**). Upon optimization, conversion of cinnamaldehyde (**3**) to its reduced counterpart (**5**) was found to be >90% (Table 1). This work lays the basis for enabling organocatalytic transfer hydrogenation in biological contexts.

**Fig. 1:**
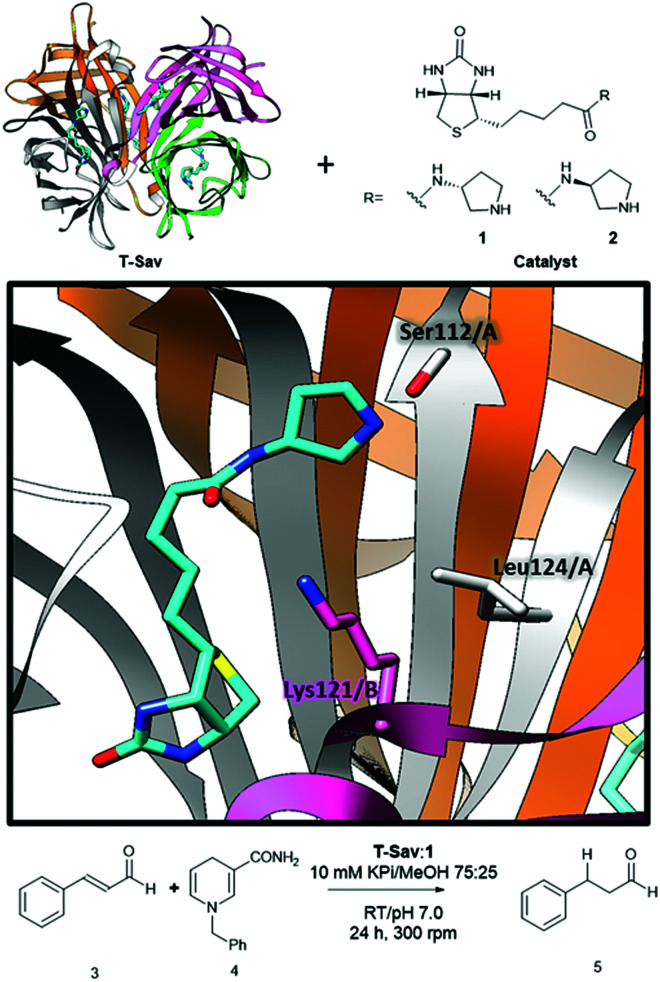
Combining the tetrameric streptavidin (**T-Sav**) with biotinylated organocatalysts **1** or **2** to generate organocatalytic artificial enzymes (**T-Sav**:**1**, PDB: 6GH7, 1.08 Å) for transfer hydrogenation reactions.

**Table 1:** Screening reaction for transfer hydrogenation from BNAH to cinnamaldehyde^a^^b^^c^

Entry	Host	Guest	Hydrogen donor	Loading (mol%)	% Estimated conversion^a^
1	N.A.	N.A.	BNAH (**4**)	N.A.	3
2	**T-Sav**	N.A.	BNAH (**4**)	1	5
3	**M-Sav**	N.A.	BNAH (**4**)	1	4
4	N.A.	**1**	BNAH (**4**)	1	26
5	N.A.	**2**	BNAH (**4**)	1	25
6	**T-Sav**	**1**	BNAH (**4**)	1	77
7	**T-Sav**	**1**	BNAH (**4**)	2	95
8	**T-Sav**	**1**	BNAH (**4**)	1	94^d^
9	**T-Sav**	**1**	BNAH (**4**)	1	53^e^^,^^f^
10	**T-Sav**	**2**	BNAH (**4**)	1	51
11	**M-Sav**	**1**	BNAH (**4**)	1	21
12	**M-Sav**	**2**	BNAH (**4**)	1	10
13	**T-Sav**	**1**	NADH (**5**)	1	5
14	**T-Sav**	**1**	Hantzsch (**6**)	1	0

a Estimated conversion was determined by the ratio of the corresponding ^1^H NMR peak integration (see ESI).

b Reactions performed in a mixture 90 : 10 KPi 10 mM : MeOH at pH 7.0 using 1 equivalent of aldehyde (6.6 mM) and 2 equivalents of **4** (13.2 mM).

c In the absence of catalyst, partial oxidation of BNAH was observed, but the oxidation rate is negligible when compared to the protein-hosted organocatalytic reaction.

d Reactions performed in a mixture 75 : 25 KPi 10 mM : MeOH at pH 7.0 using 1 equivalent of aldehyde (6.6 mM) and 5 equivalents of **4** (33 mM).

e 0 rpm, side-product observed.

f ppt observed. 1 mol% of **T-Sav**/**M-Sav** corresponds to 66 nmol catalytic sites. N.A. indicates not added.

In previous studies, we have demonstrated that biotinylated catalysts (**1** and **2**) were able to mediate Michael addition and aldol condensation when introduced to the tetrameric streptavidin (**T-Sav**).^31^ In this work, the reduction of cinnamaldehyde **3** by dihydrobenzyl nicotinamide (BNAH, **4**) in KP_i_ buffer (10 mM, 10% methanol, pH 7.0) at room temperature serves as the model transfer hydrogenation reaction and was assessed by ^1^H NMR spectroscopy (Table 1). In the absence of protein or catalysts, only 3% of dihydrogenated product was observed after 24 hour incubation when two equivalents of BNAH were used (entry 1). Similarly, **T-Sav** (and its monomeric counterpart **M-Sav**^32,33^) has negligible effects in catalyzing hydride transfer (entries 2 and 3). When 1 mol% of catalyst **1** or **2** is used, the estimated reaction conversion increased to about 25% (entries 4 and 5). When both **T-Sav** and catalyst **1** are included (1 : 1.2 ratio of catalyst to protein), the estimated conversion increased to 77 and 95% at 1 and 2 mol% respectively (entries 6 and 7). These results are in line with the findings that organocatalysis is favored when executed in an environment that has low dielectric constant, such as organic solvents and protein surface or interior.^19^ Increasing the amount of BNAH to 5 equivalents in a 75 : 25 KPi : MeOH mixture enhances the conversion up to 94% with only 1 mol% of **T-Sav**:**1** (entry 8). Interestingly, when no shaking was applied to the reaction mixture, the product conversion decreases to 53% (entry 9). Lastly, in agreement with the previous studies which illustrated that catalyst **2** is more flexible and solvent-exposed when bound to **T-Sav**,^20^ the conversion was lower when catalyst **2** was used (entry 10).

In most streptavidin-based artificial enzymes, the catalytic centers are surrounded by residues at the intersubunit interface.^24,34^ It however has recently been demonstrated that such a shielded environment might not be ideal for some of the chemical catalytic reactions; indeed, the monomeric variant **M-Sav** can be a superior host to **T-Sav** for Rh-catalyzed reactions.^33^**M-Sav** was thus tested as an alternative host for the organocatalytic transfer hydrogenation reaction. Nevertheless, in this case the conversion dropped to 21% and 10% by catalyst **1** and **2** in **M-Sav**, respectively (entries 11 and 12). This observation suggests that secondary amine-catalyzed transfer hydrogenation prefers a more shielded protein scaffold. To see how a change in hydride donor affects the conversion rate, alternative hydride donors including Hantzsch ester (**5**) and NADH (**6**) were tested, but their use resulted in significantly lower conversion (entry 13 and 14). The negligible conversion observed with NADH (**5**) could be caused by its structural complexity, whereas the low solvent solubility of Hantzsch ester (**6**) in aqueous buffer can influence its ability to transfer hydride within the **T-Sav** scaffold.

Having the optimal conditions determined (Table 1, entry 8), various aromatic α,β-unsaturated aldehydes were tested as alternative substrates. The **T-Sav**:**1** assembly showed significant substrate promiscuity; cinnamaldehyde analogues with chloro, fluoro or nitro substituent added at the *para* position can be converted into the corresponding dihydro-products (Table 2), while the background reactions remain negligible. Supported by NMR analysis, modest conversion was also observed for the methyl, bromo and methoxy analogues (60–76%). Together, these observations suggest that the efficiency of hydride transfer is largely affected by electrostatic properties of the substituents.

**Table 2:** Substrate scope for transfer hydrogenation from BNAH to aromatic α,β-unsaturated aldehydes mediated by **T**-**Sav**:**1** assembly^a^

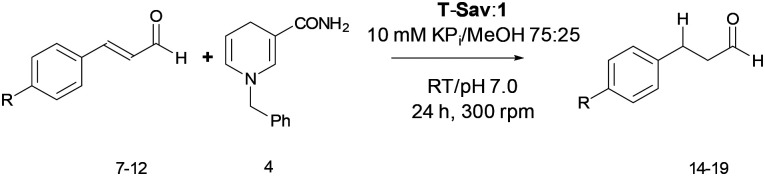	
Aldehyde	% Estimated conversion^b^
Cinnamaldehyde (**3**)	94
*p*-Cl (**8**)	94
*p*-F (**9**)	>99
*p*-Br (**10**)	60
*p*-OCH_3_ (**11**)	69
*p*-NO_2_ (**12**)	>99^c^
*p*-CH_3_ (**13**)	76

a Estimated conversion was determined by ratio of the corresponding ^1^H NMR peak integration (see ESI).

b Reactions performed in a mixture 75 : 25 KPi 10 mM : MeOH at pH 7.0 using 1 equivalent of aldehyde (6.6 mM) and 5 equivalents of **4** (33 mM) with 1 mol% of **T-Sav**:**1**.

c Reaction performed in a mixture of 9 : 1 KPi 10 mM : MeOH at pH 7.0 using 1 equivalent of aldehyde (6.6 mM) and 2 equivalents of **4** (13.2 mM). 1 mol% of **T-Sav**/**M-Sav** correspond to 66 nmol catalytic sites.

Kinetic properties of the streptavidin-based transfer hydrogenation system were evaluated. The protein-based catalyst demonstrates enzyme-like kinetic behaviours with catalytic efficiency (*k*_cat_/*K*_M_^cinnamaldehyde^) estimated to be 8.50 ± 0.5 M^−1^·s^−1^ at 25 °C (Fig. 2). The corresponding *k*_cat_/*K*_M_ for flavoprotein was reported to be 5900 M^−1^·s^−1^. Furthermore, BNAH has been used for aromatic hydroxylation and the reduction of the α,β-unsaturated carbonyl substrate; their biomolecular reaction rate constants were measured to be 1.2·10^4^ and 1.9·10^6^ M^−1^·s^−1^, respectively.^13,14^ Although these values are significantly higher than that of the streptavidin system, the presented bottom-up approach has not yet been engineered and offers significant flexibility in design.

**Fig. 2:**
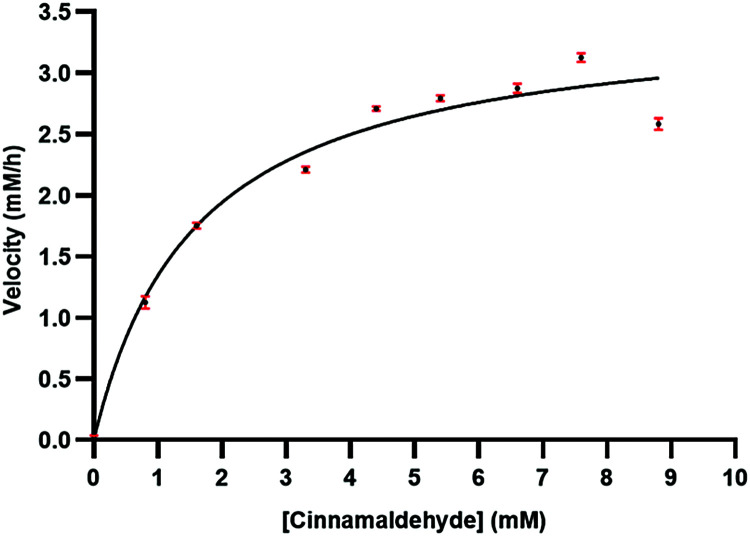
Kinetic evaluation of the **T-Sav**:**1** complex for transfer hydrogenation from BNAH to cinnamaldehyde.

QM/MM molecular dynamics (MD) simulations were conducted to obtain a detailed understanding of the hydride transfer step in the **T-Sav**:**1** system. Exploration of the free energy surface at M06-2X:RM1/MM level enabled identification of the isolated structures of the reactants (in solution as solvated species and in the active site of the protein scaffold as the “Michaelis complex”) and the hydride transfer transition state (see ESI,† for details). Hence, the magnitude of the [1,2-^2^H/^1^H] KIE was computationally assessed, including quantum tunneling corrections required for chemical reactions involving the transfer of a light particle such as hydride transfer. The resulting KIE was found to be 3.89 ± 0.15 when the ground state of BNAH in aqueous solution was used, and this parameter further increased to 4.28 ± 0.18 when the equilibrium between the Michaelis complex and the hydride transfer transition state were used for calculation (see ESI,† for details). These values are similar to those previously obtained for hydride transfer processes catalyzed by NADH-dependent enzymes,^35,36^ including l-lactate dehydrogenase (in the range between 3.36 and 2.80)^37^ and morphinone reductase (8.4 ± 1.6),^38^ but significantly higher than that of the experimental counterpart measured in the present study. Interestingly, regarding the hydride donor–acceptor distance and the relative orientation of the different involved moieties, the nature of the optimized transition state can be considered as equivalent to those located in previous studies (Fig. 3).^35–38^ It therefore suggests that the hydride transfer step in the **T-Sav** artificial enzyme is at least partially rate-limiting.

**Fig. 3:**
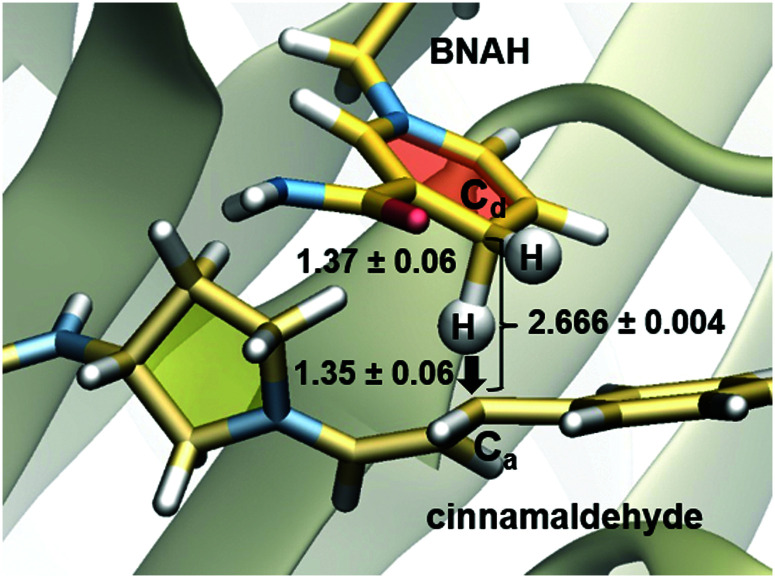
Detail of the transition state for the hydride transfer from BNAH to cinnamaldehyde in the active site of **T-Sav**:**1** optimized at the M06-2X/MM level. Average distances in Å.

In summary, we have described the use of streptavidin as a host for organocatalytic transfer hydrogenations. At 1 mol%, the **T-Sav**:**1** assembly was able to mediate the hydrogenation of various aromatic α,β-unsaturated aldehydes in excellent conversions, and the resulting products have been used in the synthesis of pharmaceutical compounds.^39^ Moreover, **T-Sav**:**1** possesses Michaelis Menten kinetic properties for the organocatalytic transfer hydrogenation, presenting as a great starting point for artificial enzyme engineering. Studies focusing on the reaction mechanisms, the use of prochiral aldehydes and potential applications in cascade reactions are currently ongoing.

This work was supported by Cardiff University through the start-up fund provided by the Cardiff School of Chemistry, the Leverhulme Trust through a grant to L. Y. P. L. (RPG-2017-195), the Royal Society through a grant to L. C. M. (RG150466), and the UK's Wellcome Trust through grants to L. Y. P. L. (202056/Z/16/Z).

## Conflicts of interest

There are no conflicts to declare.

## Supplementary Material

CC-057-D0CC08142F-s001
