# Supplementary material for: Transfer hydrogenations catalyzed by streptavidin-hosted secondary amine organocatalysts
Source: Chem Commun (Camb). 2021 Jan 14;57(15):1919–22. doi: 10.1039/d0cc08142f (PMC8330412; doi:10.1039/d0cc08142f)
Supplement: CC-057-D0CC08142F-s001 [file CC-057-D0CC08142F-s001.pdf]

Electronic Supplementary Material (ESI) for ChemComm.
This journal is © The Royal Society of Chemistry 2021

Supporting Information

Transfer Hydrogenations catalyzed by Streptavidin-hosted Secondary Amine Organocatalyst

Nicolò Santi,[a] Louis C. Morrill,[a,b] Katarzyna Świderek,[c] Vicent Moliner,[c] Louis Y. P. Luk*,[a,b]

[a] School of Chemistry, Main Building, Cardiff University, Cardiff, CF10 3AT (UK)
[b] Cardiff Catalysis Institute, School of Chemistry, Main Building, Cardiff University, Cardiff, CF10 3AT (UK)
[c] Departament de Química Física i Analítica, Universitat Jaume I, 12071 Castelló, (Spain)
*E-mail: lukly@cardiff.ac.uk

Table of Contents

1 General Information 4

2 Experimental Details for the Preparation and Purification of M-Sav 6

2.1 Expression and Purification 6

2.2 Plasmid map M-Sav 7

3 Experimental Details for the Activity Screening of Catalysts 1 and 2 and T-Sav in the Transfer Hydrogenation from BNAH to Cinnamaldehyde 8

3.1 ^{1}H NMR Based Screening for Yield Determination 8

3.2 Exemplary ^{1}H NMR Spectrum after Extraction of the Biphasic Reaction Mixtures 8

4 Experimental Details for the Synthesis..10

4.1 Synthesis of *N*-benzyl-3-carbamoylpiridinium bromide (4a) 10

4.2 Synthesis of *N*-benzyl-1,4-dihydronicotinamide (BNAH) (4)...10

5 ^{1}H NMR and HRMS Details for compounds synthesised 11

5.1 ^{1}H NMR details for *N*-benzyl-3-carbamoylpyridin-1-ium bromide (4a) 11

5.2 ^{1}H NMR details for *N*-benzyl-1,4-dihydronicotinamide (BNAH, 4) 12

6 ^{1}H NMR Details for the Activity Screening of different catalysts for the Hydride Transfer of several hydride donors to A, B-unsaturated aldehydes 13

6.1 Catalyst Screening Reactions for the Hydride Transfer of BNAH to Cinnamaldehyde 13

6.1.1 No Catalyst...13

6.1.2 Catalyst 1 14

6.1.3 Catalyst 2 15

6.1.4 T-Sav 16

6.1.5 M-Sav...17

6.1.6 T-Sav:1 18

6.1.7 T-Sav:2 19

6.1.8 M-Sav:1..20

6.1.9 M-Sav:2..21

6.1.10 T-Sav:1, no shaking (0 rpm) 22

6.1.11 T-Sav:1 (5 equivalents BNAH, 25% MeOH) 23

6.1.12 T-Sav:1, 2 mol% 24

6.2 Hydride Donors Screening Reactions for the Hydride Transfer to Cinnamaldehyde.................25

6.2.1 NADH 25

6.2.2 Hantzsch ester 26
6.3 Substrate Scope for the Hydride Transfer from BNAH to Different A, B-Unsaturated Aldehydes (5 Equivalent BNAH, 25% Methanol as Co-Solvent) 27
6.3.1 (*E*)-3-(*p*-chlorophenyl) acrylaldehyde 27
6.3.2 (*E*)-3-(*p*-fluorophenyl) acrylaldehyde 28
6.3.3 (*E*)-3-(*p*-bromophenyl) acrylaldehyde 29
6.3.4 (*E*)-3-(*p*-methoxyphenyl) acrylaldehyde 30
6.3.5 (*E*)-3-(*p*-nitrophenyl) acrylaldehyde 31
6.3.6 (*E*)-3-(*p*-tolyl) acrylaldehyde 32
7 Michaelis-Menten Kinetic for the Reaction Between 3 and 4 33
7.1 Procedure for the Kinetic Assessment 33
8 Computational methods. 34
8.1 Computational details of the molecular models set up 34
8.2 Computational details of the QM/MM simulations 37
8.3 Potential Energy Surfaces 37
8.4 Free Energy Surfaces 38
8.5 Spline Corrections 38
8.6 Quantum tunnelling corrections 40
8.7 Isotope effects 40
9 References 42

1 General Information

Reactions were performed in oven dried glassware without precautions to exclude air. Reaction temperatures are stated as heating device temperature (e.g. oil bath, shaker, etc.), if not stated otherwise. Concentrations under reduced pressure were performed by rotary evaporation at 40°C at the appropriated pressure, unless otherwise noted. Deionized water was obtained from an *Elga PURELAB Option* system (15 MΩ·cm). Analytical and preparative thin layer chromatography (TLC) was carried out with silica gel 60 F254 aluminium sheets from *Merck*. Detection was carried out using UV light (λ = 254 nm and 366 nm), followed by immersion in permanganate staining solution with subsequent development via careful heating with a heat gun. Flash column chromatography was performed using silica gel (pore size 60 Å, 0.040-0.063 mm).

Cinnamaldehyde (**3**) for transfer hydrogenation reactions with **T-Sav** and **M-Sav** is commercially available and was used without further purification. For all other reactions, it was washed aqueous sodium bicarbonate solution (pH 8.3), dried over magnesium sulphate and stored under inert atmosphere at –23 °C. Nicotinamide adenine dinucleotide (NADH, **6**), diethyl 1,4-dihydro-2,6-dimethyl-3,5-pyridinedicarboxylate (Hantzsch ester, **7**), (*E*)-3-(*p*-chlorophenyl) acrylaldehyde (**8**), (*E*)-3-(*p*-fluorophenyl) acrylaldehyde (**9**), (*E*)-3-(*p*-bromophenyl) acrylaldehyde (**10**), (*E*)-3-(*p*-methoxyphenyl) acrylaldehyde (**11**), (*E*)-3-(*p*-nitrophenyl) acrylaldehyde (**12**), (*E*)-3-(*p*-tolyl) acrylaldehyde (**13**), 3-phenyl propanal (**14**) and (*S*)-α,α-Bis[3,5-bis(trifluoromethyl)phenyl]-2-pyrrolidinemethanol trimethylsilyl ether (**20**) are commercially available and were used without further purification. All other solvents and reagents were obtained from commercial sources and used as received.

The biotinylated organocatalysts **1** and **2** were synthesised as previously reported.[1]

T-Sav (Streptavidin *Streptomyces avidinii* recombinant, tetramer, *MW* ≈ 52 kDa, "core" Streptavidin with amino acids 13-139) was obtained commercially from *ProSpec* (PRO-791) as lyophilized powder in 10 mM KP_i pH 6.5 and stored at −23 °C upon receipt until further use. According to the supplier **T-Sav** has the following amino acid sequence:

```
MAEAGITGTWYNQLGSTFIVTAGADGALTGTYESAVGNAESRYVLTG
RYDSAPATDGSGTALGWTVAWKNNYRNAHSATTWSGQYVGGAEARIN
TQWLLTSGTTEANAWKSTLVGHDTFTKVKPSAAS
```

The plasmid for **M-Sav** (encoding for a monomeric Streptavidin/Avidin recombinant protein, *MW* ≈ 15.7 kDa) was obtained as a gift from Sheldon Park (pRSET-mSA, Addgene plasmid #39860, http://n2t.net/addgene:39860, RRID: Addgene_39860).[2] The gene encoding for **M-Sav** translates to the following amino acid sequence:

```
MHHHHHHSQDLASAEAGITGTWYNQSGSTFTVTAGADGNLTGQYENR
AQGTGCQNSPYTLTGRYNGTKLEWRVEWNNSTENCHSRTEWRGQYQG
GAEARINTQWNLTYEGGSGPATEQGQDTFTKVKPSAASGSDYKDDDD
K
```

A *VWR 3510* benchtop pH Meter connected to a *Jenway* micro pH electrode or a *VWR Universal* pH electrode were used for the pH adjustment of buffers and reaction mixtures employing either 1.0 M or 0.1 M sodium hydroxide or hydrochloric acid solution.

Shaking of the reactions at 25 °C was achieved using a *VWR* thermoshaker *Mini shake lite* or a *VWR Incubating Orbital Shaker*.

^{1}H and ^{13}C NMR spectra were recorded in $CDCl_3$, methanol-d_4, DMSO-d_6, or D_2O on *Bruker Fourier 300*, *Ultrashield 400*, *Ascend 500* equipped with N_2 cryoprobe, or *Advance 600* equipped with He cryoprobe instruments. Chemical shifts are reported in parts per million (ppm) and are referenced to the residual solvent resonance as the internal standard ($CHCl_3$: δ = 7.26 ppm for ^{1}H; methanol-d_3: δ = 3.31 ppm for ^{1}H; D_2O: δ = 4.79 ppm for ^{1}H). Data are reported as follows: chemical shift, multiplicity (br s = broad singlet, s = singlet, d = doublet, dd = double doublet, td = triple doublet, t = triplet, dt = double triplet, q = quartet, p = pentet, sept = septet, br m = broad multiplet, m = multiplet, m_c = centrosymmetric multiplet), coupling constants (Hz) and integration.

High resolution mass spectra (HRMS) were recorded on a *Waters LCT Premier* for ESI-(+) and APCI-(+) or a *Waters GCT Premier* (EI) system.

Size exclusion chromatography was performed using a ÄKTA Purifier workstation (GE Healthcare) or a Bio-Rad system with the respective column mentioned in the detailed procedure.

2 Experimental Details for the Preparation and Purification of M-Sav

2.1 Expression and Purification

Monomeric streptavidin (**M-Sav**) was expressed using an *E. coli* expression system with the following protocol. Plasmid pRSET-mSA (see 2.2) containing the desired **M-Sav** gene (**Fig. S1**) in a pRSET-A vector was transformed into calcium competent BL21 AI cells and grown for 16 h on LB agar plates containing 100 µg/mL ampicillin. A single colony from the plate was picked to inoculate a 5 mL LB culture for overnight. The starter culture was diluted into 500 mL of LB medium containing 100 µg/mL ampicillin. The culture was grown at 37 °C and 225 rpm to an OD_{600} of 0.8−1.0 and induced with a final concentration of 0.5 % w/v L-arabinose. The culture was grown overnight at 20 °C. The pellet was harvested by centrifugation at 4000 rpm at 4 °C for 25 min, resuspended in 10 mL of wash buffer (50 mM Tris-HCl, 100 mM NaCl, pH 8.0) and lysed via sonication (7 min, 5 sec on, 10 sec off). 10 mL of lysate buffer (50 mM Tris-HCl, 100 mM NaCl, and 6 M GdnHCl, pH 8) were added to the suspension and left to incubate for 3 h at 4 °C. The insoluble fraction was removed by centrifugation at 4000 rpm at 4 °C for 25 min, and the supernatant was mixed with 3 mL of Ni-NTA affinity resin. After incubation at 25 °C for 1.5 h with occasional stirring, the resin was washed twice with 1.5 volumes of resin wash buffer (50 mM Tris-HCl, 100 mM NaCl, 6 M GdnHCl, and 10 mM imidazole, pH 7.5). **M-Sav** was eluted with elution buffer (3× 3.5 mL: 50 mM Tris-HCl, 150 mM NaCl, 6 M GdnHCl, 300 mM imidazole, pH 8.0). Samples of the wash and elution fractions were collected and analysed by SDS-PAGE (15% w/v). The elution fractions were added drop by drop to 40 mL of ice-cold refolding buffer (50 mM Tris-HCl, 150 mM NaCl, 0.3 mg/mL catalyst **1** or **2**, 0.2 mg/mL oxidized glutathione, and 1 mg/mL reduced glutathione) under rapid stirring to refold the protein. The precipitates were removed by centrifugation at 4000 rpm at 4 °C for 25 min. The refolded protein solution was concentrated to 5 mL using Amicon ultra centrifugation with a 10 kDa cut-off. The concentrated protein solution was applied to size exclusion chromatography (Hi-Load^{TM} column, Superdex 200 pg, 50 mM Tris-HCl, 150 mM NaCl, pH 8.0). Fractions containing protein (analysis by following 215, 255 and 280 nm UV traces) were collected and the samples loaded on SDS-PAGE (15% w/v) to check the purity of the protein. Fractions containing **M-Sav** were pooled, transferred to a centrifugal concentrator with a 10 kDa cut-off and the buffer was exchanged to 10 mM KP_i, pH 7.0.

2.2 Plasmid map M-Sav

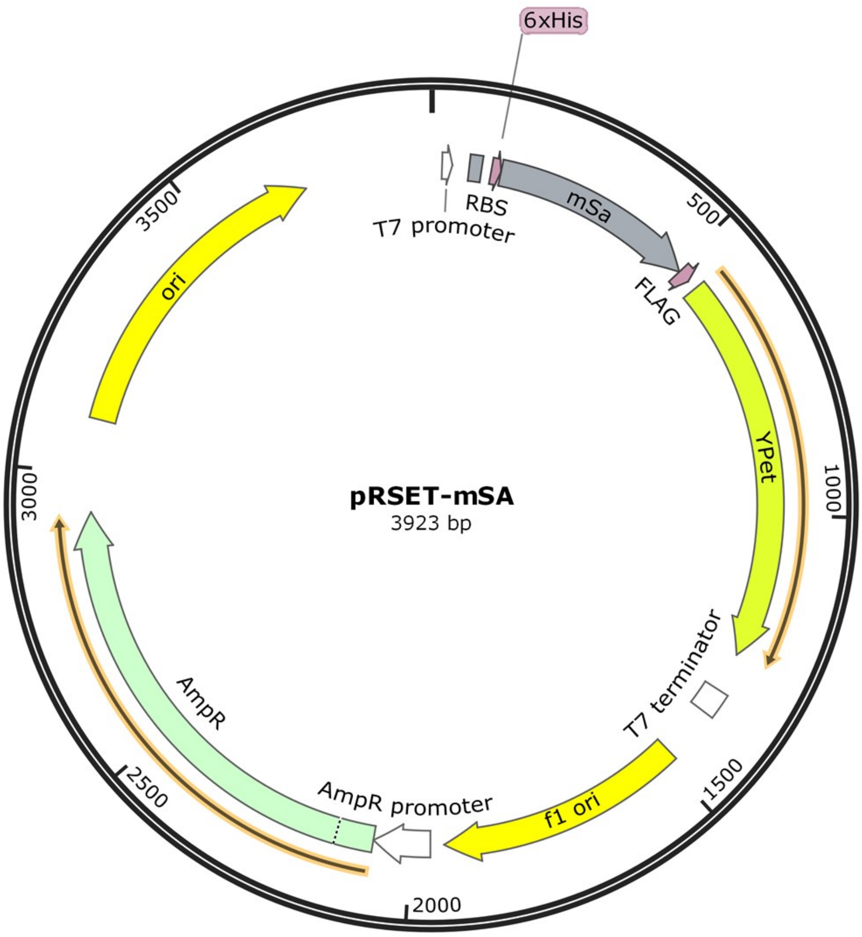

Fig. S1. Plasmid map for the **M**-**Sav** gene.

3 Experimental Details for the Activity Screening of Catalysts 1 and 2 and T-Sav in the Transfer Hydrogenation from BNAH to Cinnamaldehyde

3.1 ^{1}H NMR Based Screening for Yield Determination

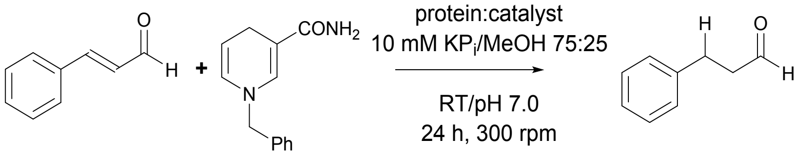

3 4 5

Scheme S1. Transfer hydrogenation from BNAH to cinnamaldehyde mediated by different protein:host assemblies.

To the respective **T-Sav**:**1/2** or **M-Sav**:**1/2** solution (250 µL, 2.32 mg/mL, 33 nmol catalytic sites, 1.2 mol%, 10 mM KP_i buffer, pH 7.0), 44.2 µL of BNAH stock solution (0.38 M in MeOH, 16.5 µmol, 5.0 eq.), 2.5 µL of cinnamaldehyde stock solution (1.33 M in MeOH, 3.3 µmol, 1.0 eq.) and 78.27 µL MeOH were added (**Scheme S1**). The Eppendorf tube was placed in a Falcon tube, which was placed inside an incubating shaker (300 rpm, 25 °C). After 24 h, DCM (500 µL) and water (500 µL) were added, and the phases were separated. The aqueous phase was extracted (2 × 500 µL DCM), and the organic phases were combined. The volatiles were removed under reduced pressure, the residue was taken up in 700 µL $CDCl_3$, and transferred to an NMR tube. This was directly subjected to ^{1}H NMR analysis (See section 3.2). Each **T-Sav**:**1/2** or **M-Sav**:**1/2** catalysis were run in triplicates. This procedure was also applied for the aldehydes (**8-13**) screening. For the reactions where 5 equivalents of BNAH and 25% methanol were employed, adjustment in the calculation of BNAH and **T-Sav** stock solutions were made.

3.2 Exemplary ^{1}H NMR Spectrum after Extraction of the Biphasic Reaction Mixtures

The conversion was estimated by integration of the substrate (red box, **doublet**) and the product (yellow box, **singlet**) aldehydic peaks (**Fig. S2**, region between 9-10 ppm). Formation of the product was also confirmed by the presence of the H_α and H_β peaks (yellow box, region between 2.5-3.5 ppm). Exemplary of the crude of reaction for the reduction of cinnamaldehyde (**3**) to hydrocinnamaldehyde (**5**) in presence of BNAH (**4**) is shown in the green spectrum. The same methodology was applied to calculate the estimated conversion for all the other substrates (**8-13**). Further information is listed in section 6.

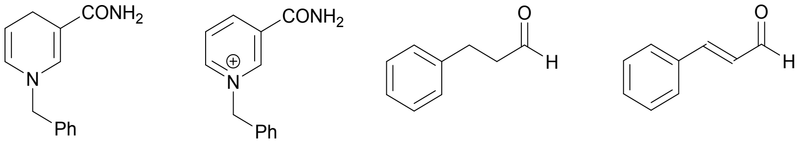

4 4a 5 3

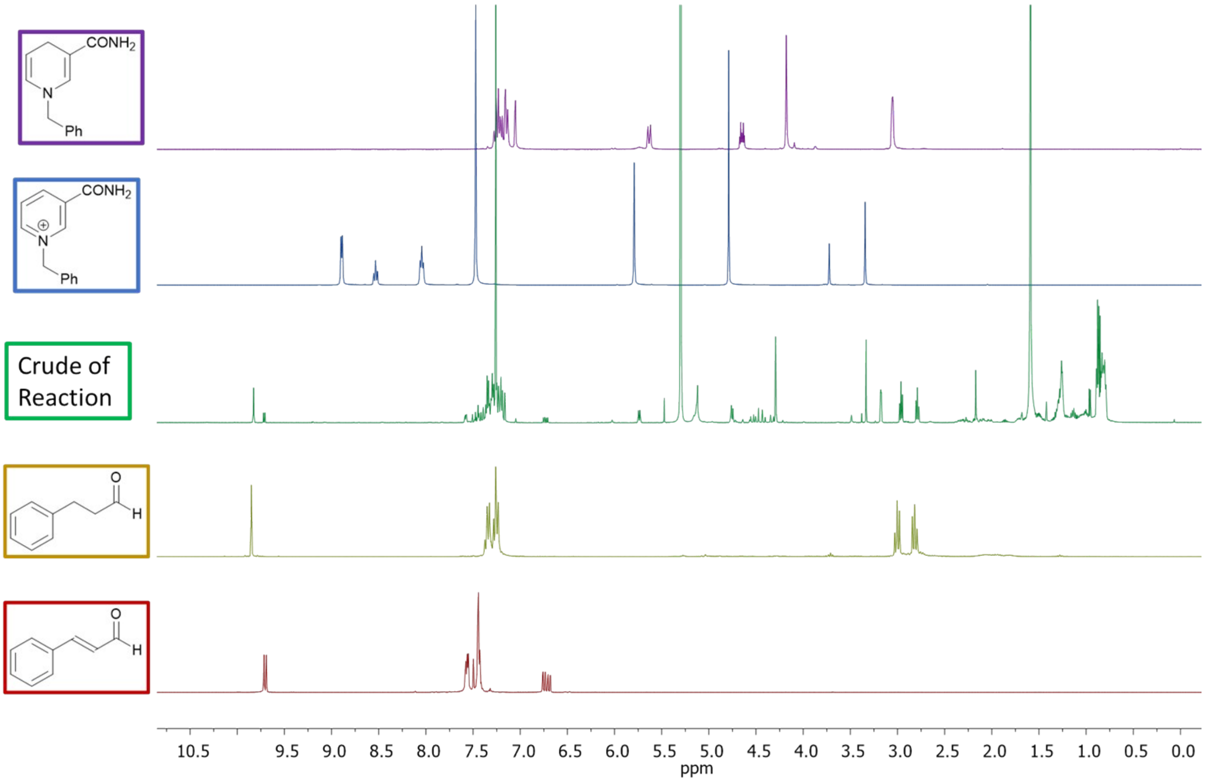

Fig. S2. ^{1}H NMR spectrum of **T**-**Sav** catalysed transfer hydrogenation. In each row are reported the spectrum of the pure compounds **4**, **4a**, **5** and **3**. In the middle is reported an exemplary of crude of reaction.

4 Experimental Details for the Synthesis

4.1 Synthesis of *N*-benzyl-3-carbamoylpiridinium bromide (4a)

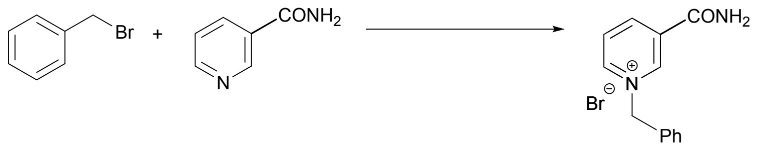

Nicotinamide (4.8 g, 1 eq., 40.00 mmol) was dissolved in 100 mL of 1,4-dioxane-methanol (4:1), and benzyl bromide (4.75 mL, 1 eq., 40.00 mmol) was added. The reaction mixture was refluxed at 80 °C for 16 h, after which time a precipitate was observed. This solution was cooled and 1,4-dioxane (50 mL) was added to further precipitate the final product. After filtration, the precipitate was washed with 1,4-dioxane (3x50 mL) and *N*-benzyl-3-carbamoylpyridinium bromide was obtained (**4a**, 6.35 g, 74.48% yield). ^{1}**H NMR** (500 MHz, D_2O): δ = 9.39 (s, 1H), 9.10 (d, *J*= 6.25 Hz, 1H), 8.94 (d, *J*= 8 Hz, 1H), 8.22 (t, *J*= 6 Hz, 1H), 7.55 (m, 5H), 5.94 (s, 2H) ppm. The analytical data is in accordance with literature.[3, 4]

4.2 Synthesis of *N*-benzyl-1,4-dihydronicotinamide (BNAH) (4)

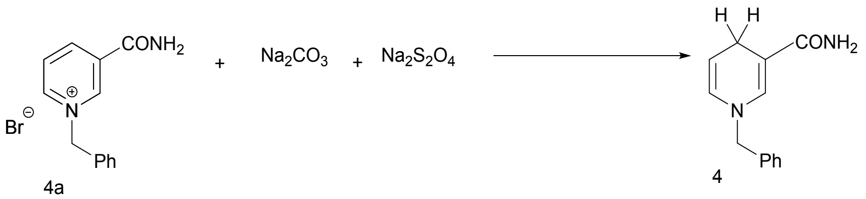

Under nitrogen atmosphere, the bromide salt of 1-benzyl-3-carbamoylpyridinium bromide (**4a**, 1.06 g, 5.00 mmol) was dissolved in distilled water (100 mL) and sodium hydrogen carbonate (2.10 g, 5 eq., 25.00 mmol) was added. Sodium dithionite (4.35 g, 5 eq., 25.00 mmol) was then added portion-wise and the reaction mixture was stirred at room temperature for 3 h in the dark. During this time, the solution turned from orange to yellow and a yellow product precipitated. The solid was filtered, washed with cold distilled water (3x50 mL) and dried over phosphorus pentoxide under vacuum to afford a bright tallow solid (**4**, 1.00 g, 93.43% yield). ^{1}**H NMR** (400 MHz, $CDCl_3$): δ = 7.35-7.30 (m, 5H), 7.16 (d, *J*= 1.6 Hz, 1H), 5.74 (dq, *J*= 8, 3.5 Hz, 1H), 5.11 (s br, 2 H), 4.75 (dt, *J*= 8, 3.5 Hz, 1H), 4.29 (s, 2H), 3.18 (dd, *J*= 3.3, 1.6 Hz, 2H) ppm. The analytical data is in accordance with literature.[3, 4]

5 ^{1}H NMR and HRMS Details for compounds synthesised

5.1 ^{1}H NMR details for *N*-benzyl-3-carbamoylpyridin-1-ium bromide (4a)

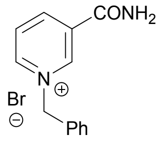

4a

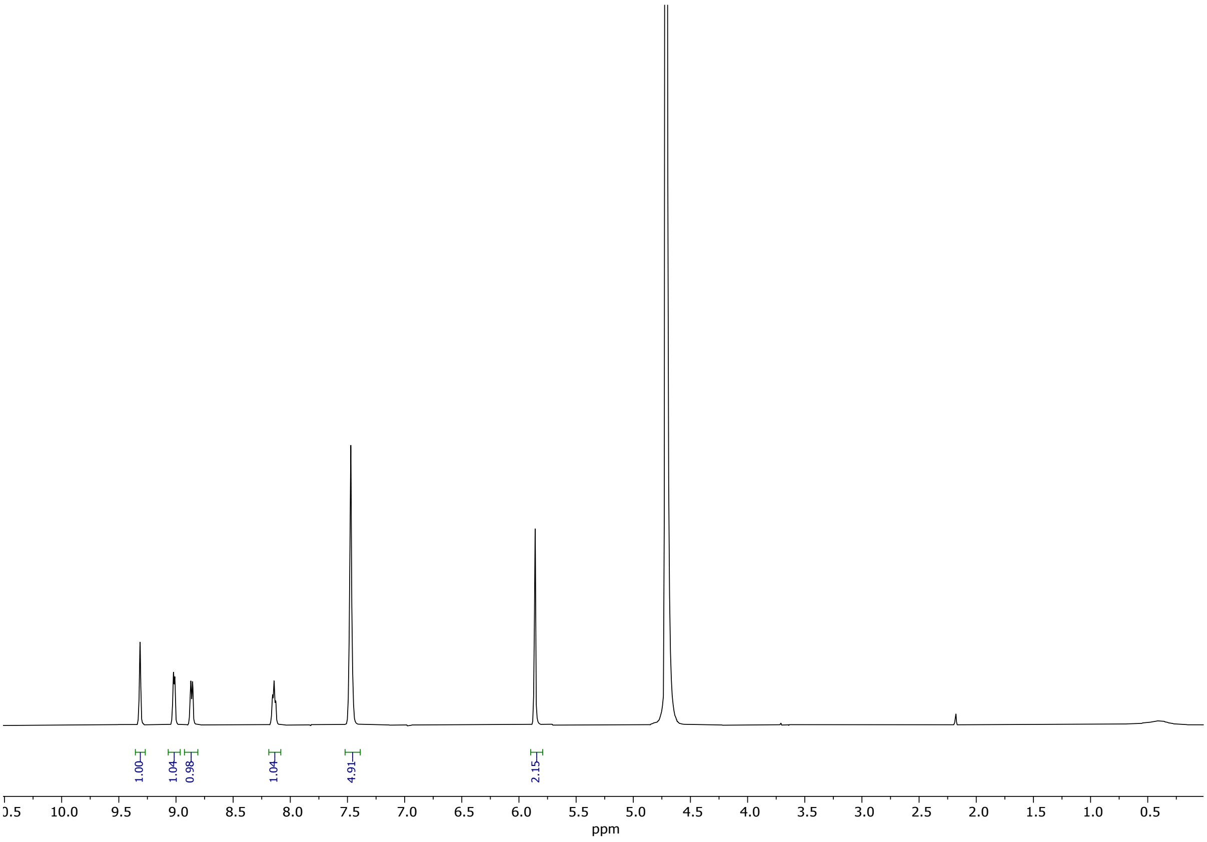

5.2 ^{1}H NMR details for *N*-benzyl-1,4-dihydronicotinamide (BNAH, 4)

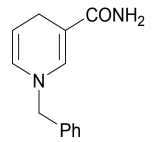

4

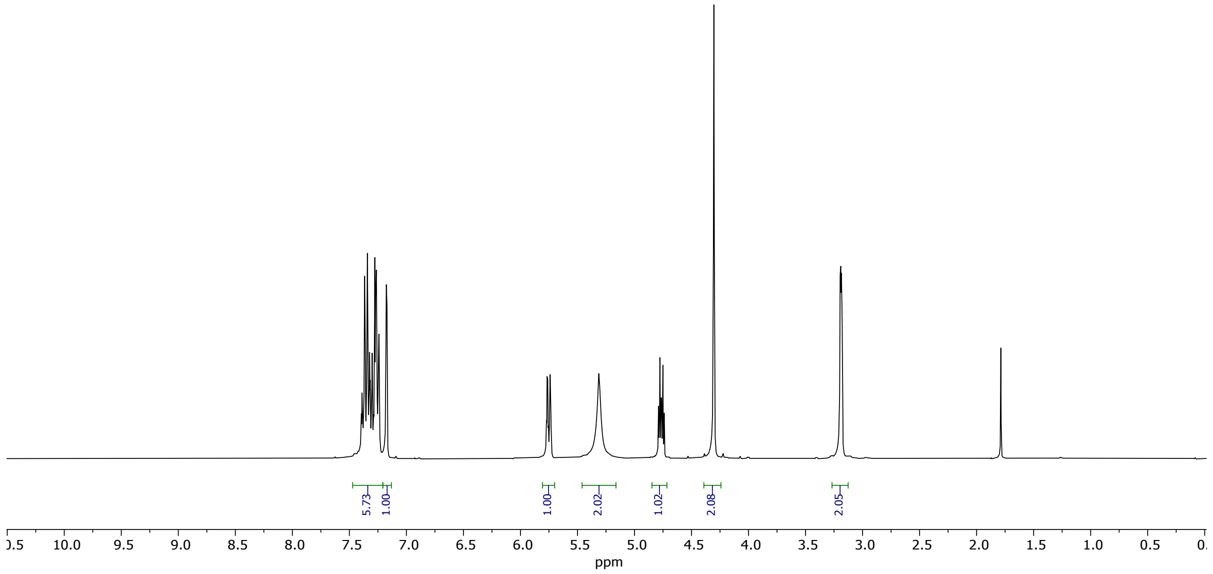

6 ^{1}H NMR Details for the Activity Screening of different catalysts for the Hydride Transfer of several hydride donors to A, B-unsaturated aldehydes

6.1 Catalyst Screening Reactions for the Hydride Transfer of BNAH to Cinnamaldehyde

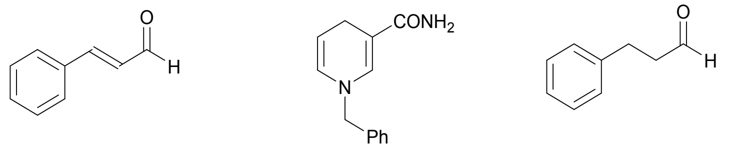

The estimated conversion of cinnamaldehyde (**3**) into hydrocinnamaldehyde (**5**) is calculated after integration of the product peak at 9.85 ppm with the substrate peak at 9.72 ppm. The spectroscopic data of **5** are the following: **^{1}H NMR** (500 MHz, $CDCl_3$): δ = 9.85 (t, *J*= 1.4 Hz, 1 H) , 7.40-7.20 (m, 5H), 3.00 (t, *J*= 7.5 Hz, 2 H), 2.82 (t, *J*= 7.5 Hz, 2 H) ppm. These data matched those reported in the literature.[6] Traces of the oxidised version of BNAH (BNA$^+$, **4a**) can be observed and confirmed by stacking the spectra of BNA$^+$ and of the crude of reaction.

6.1.1 No Catalyst

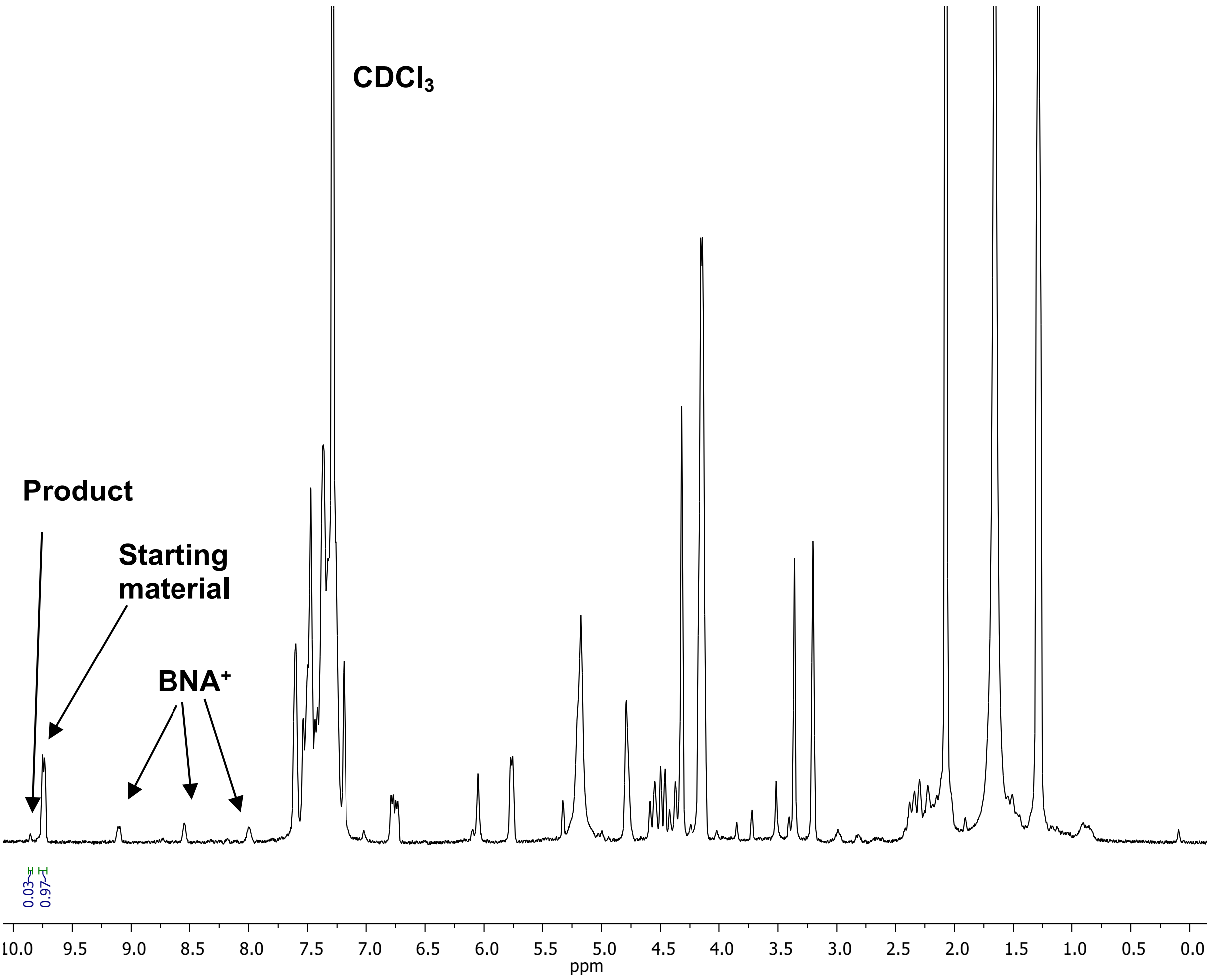

6.1.2 Catalyst 1

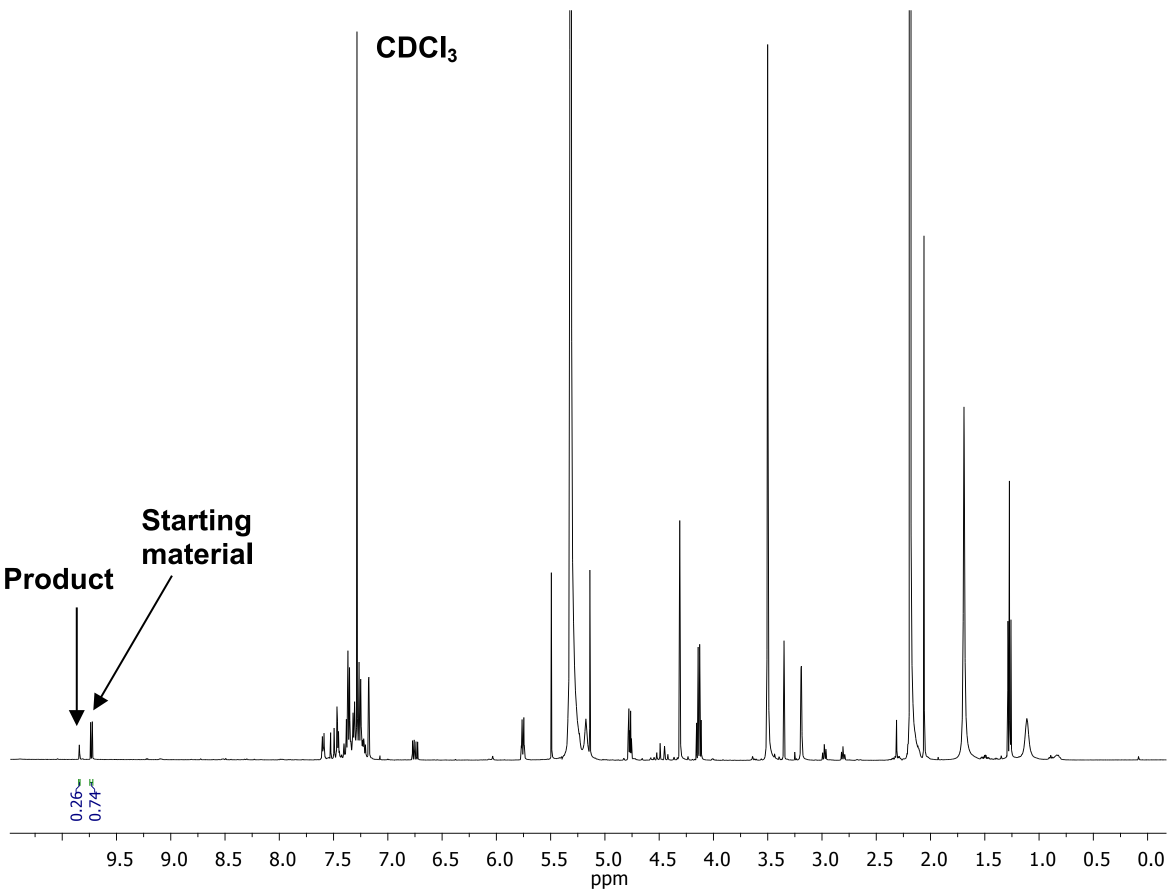

6.1.3 Catalyst 2

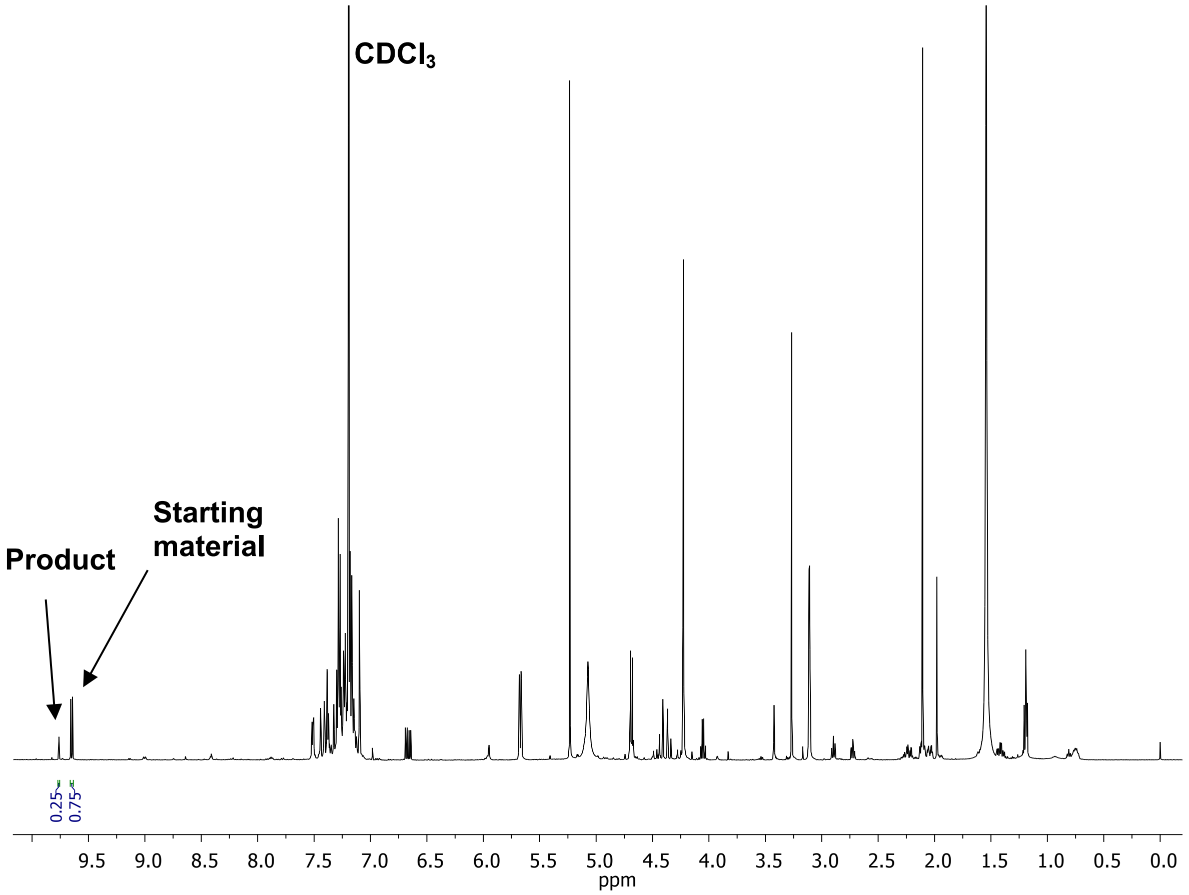

6.1.4 T-Sav

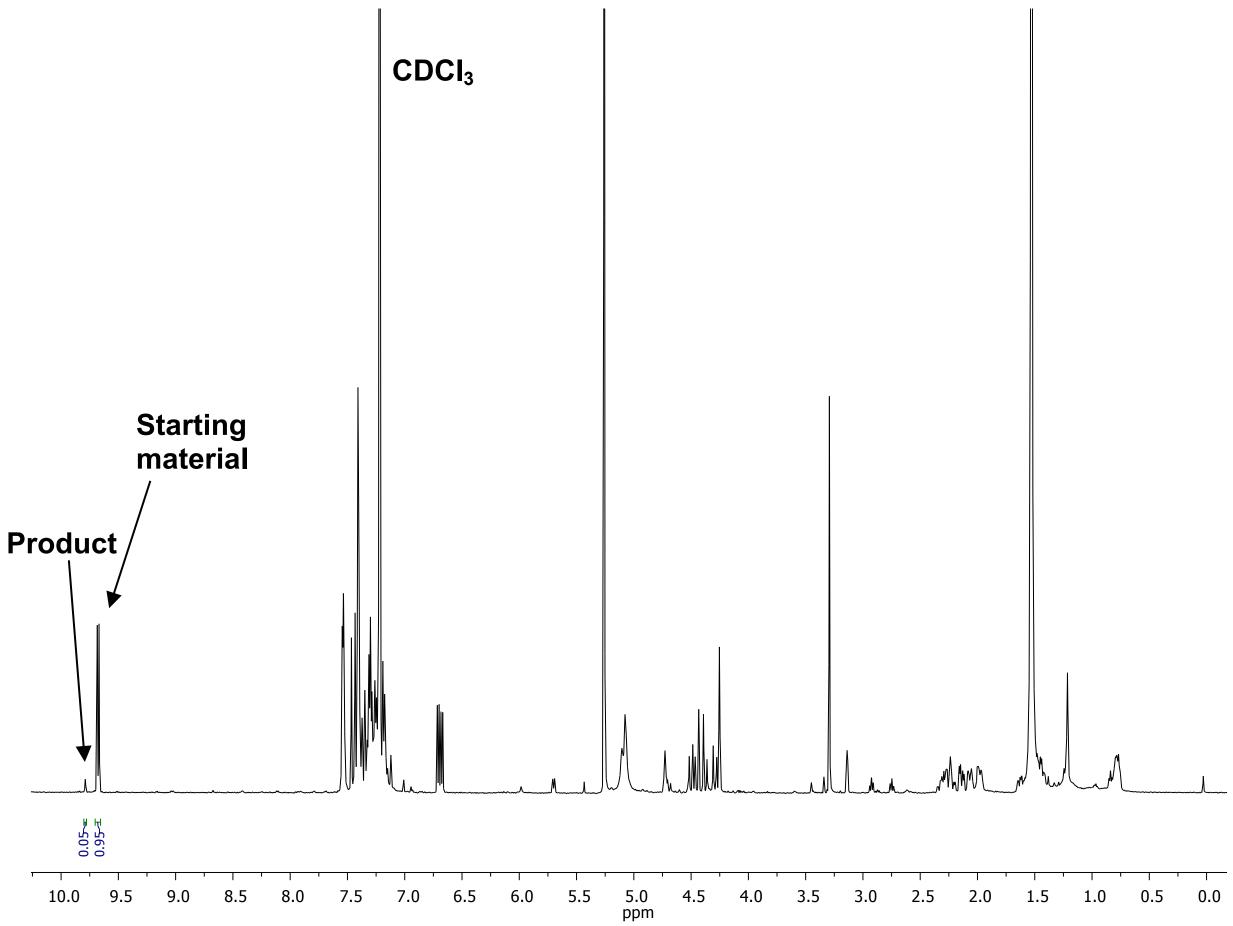
CDCl3
Starting material
Product
0.05
0.95
10.0 9.5 9.0 8.5 8.0 7.5 7.0 6.5 6.0 5.5 5.0 4.5 4.0 3.5 3.0 2.5 2.0 1.5 1.0 0.5 0.0
ppm

6.1.5 M-Sav

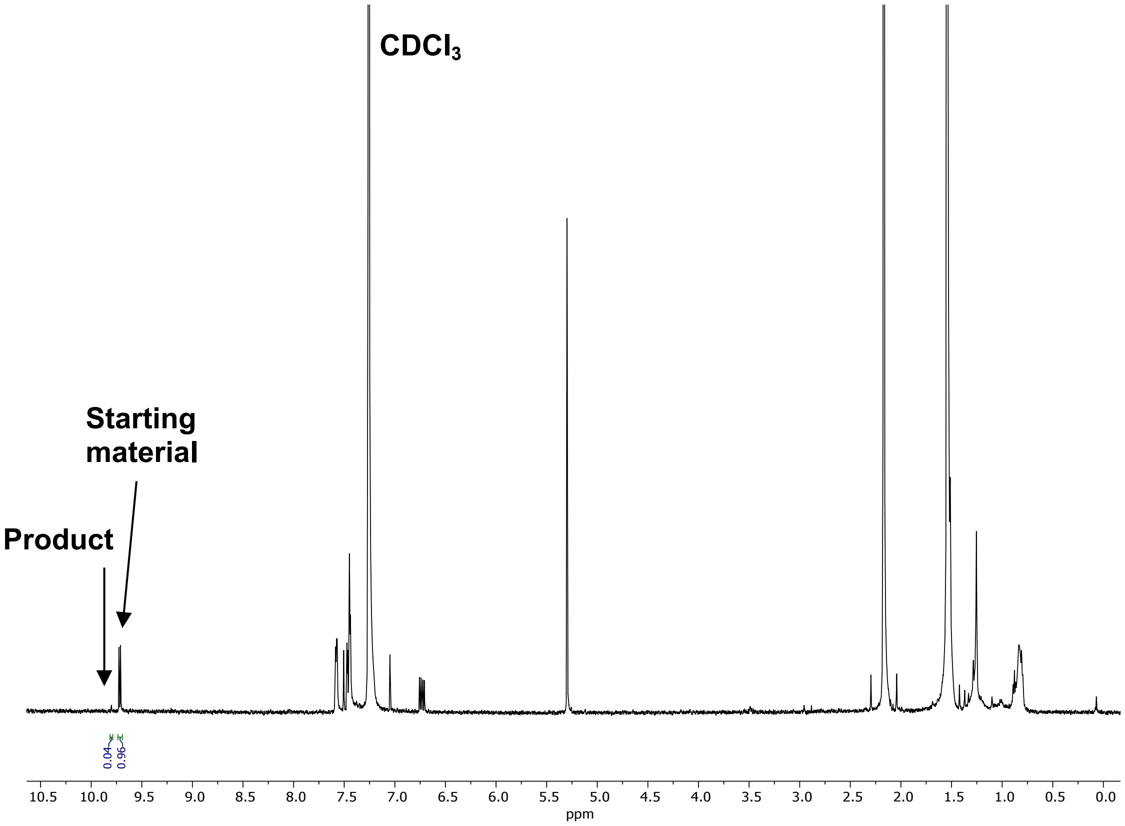

CDCl3
Starting material
Product
0.04
0.96
10.5 10.0 9.5 9.0 8.5 8.0 7.5 7.0 6.5 6.0 5.5 5.0 4.5 4.0 3.5 3.0 2.5 2.0 1.5 1.0 0.5 0.0
ppm

6.1.6 T-Sav:1

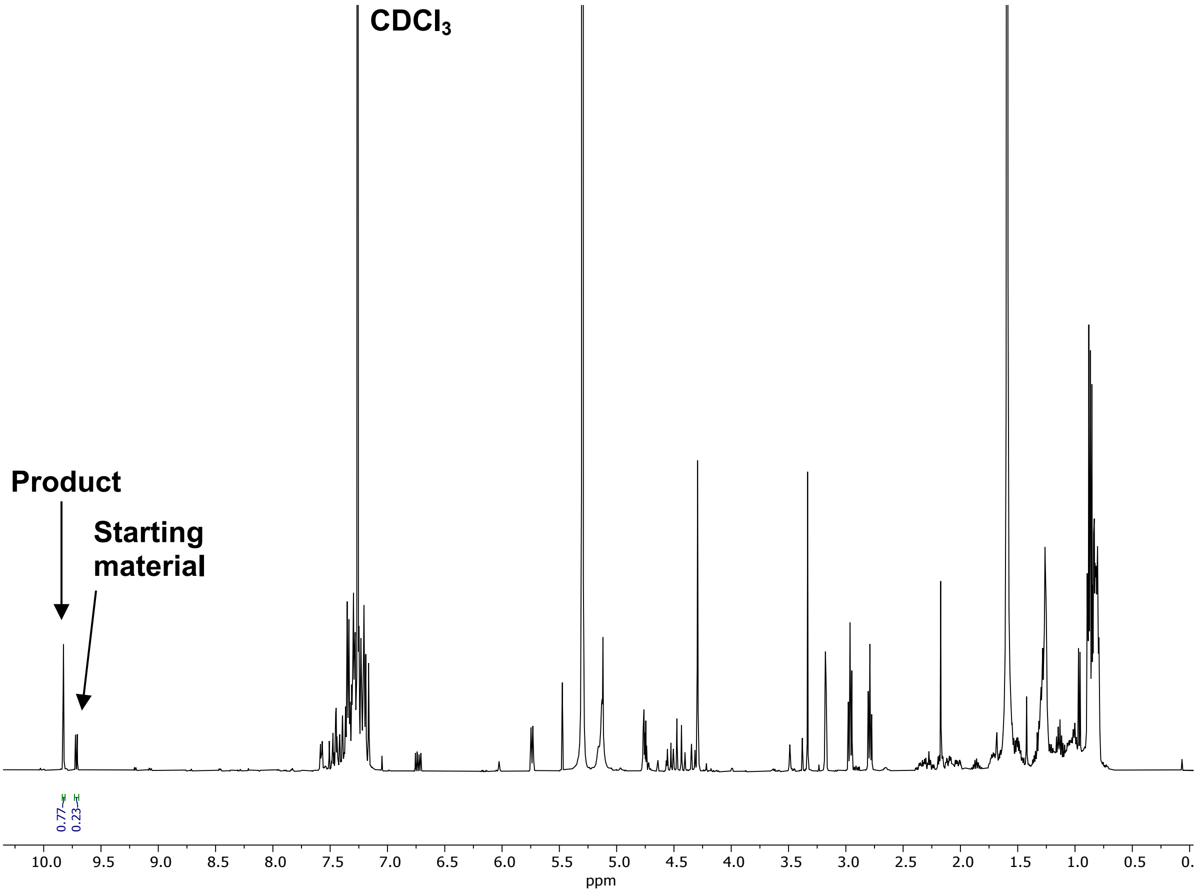
CDCl$_3$
Product
Starting material
0.77
0.23
10.0
9.5
9.0
8.5
8.0
7.5
7.0
6.5
6.0
5.5
5.0
4.5
4.0
3.5
3.0
2.5
2.0
1.5
1.0
0.5
0.
ppm

6.1.7 T-Sav:2

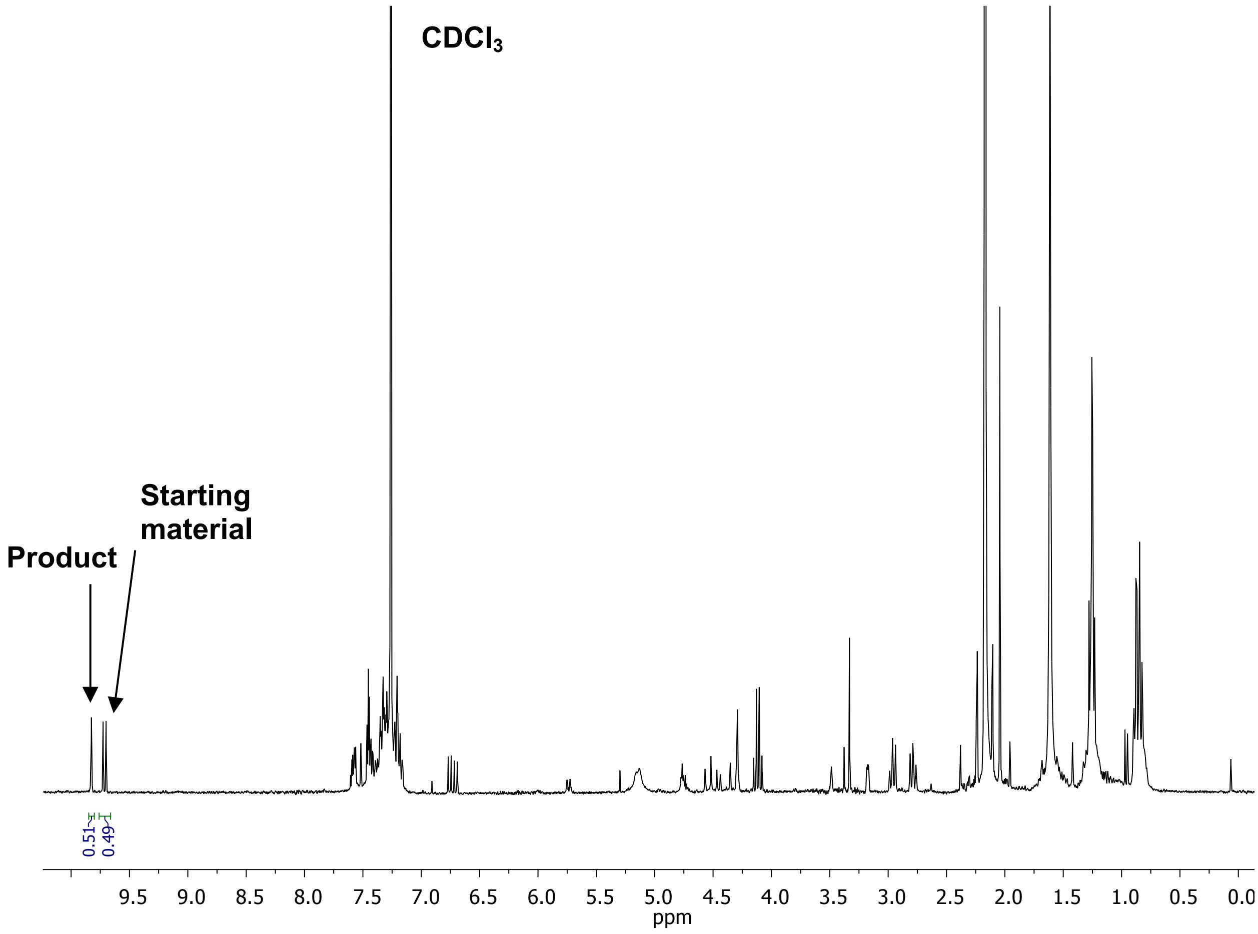

CDCl$_3$
Starting material
Product
0.51
0.49
9.5 9.0 8.5 8.0 7.5 7.0 6.5 6.0 5.5 5.0 4.5 4.0 3.5 3.0 2.5 2.0 1.5 1.0 0.5 0.0
ppm

6.1.8 M-Sav:1

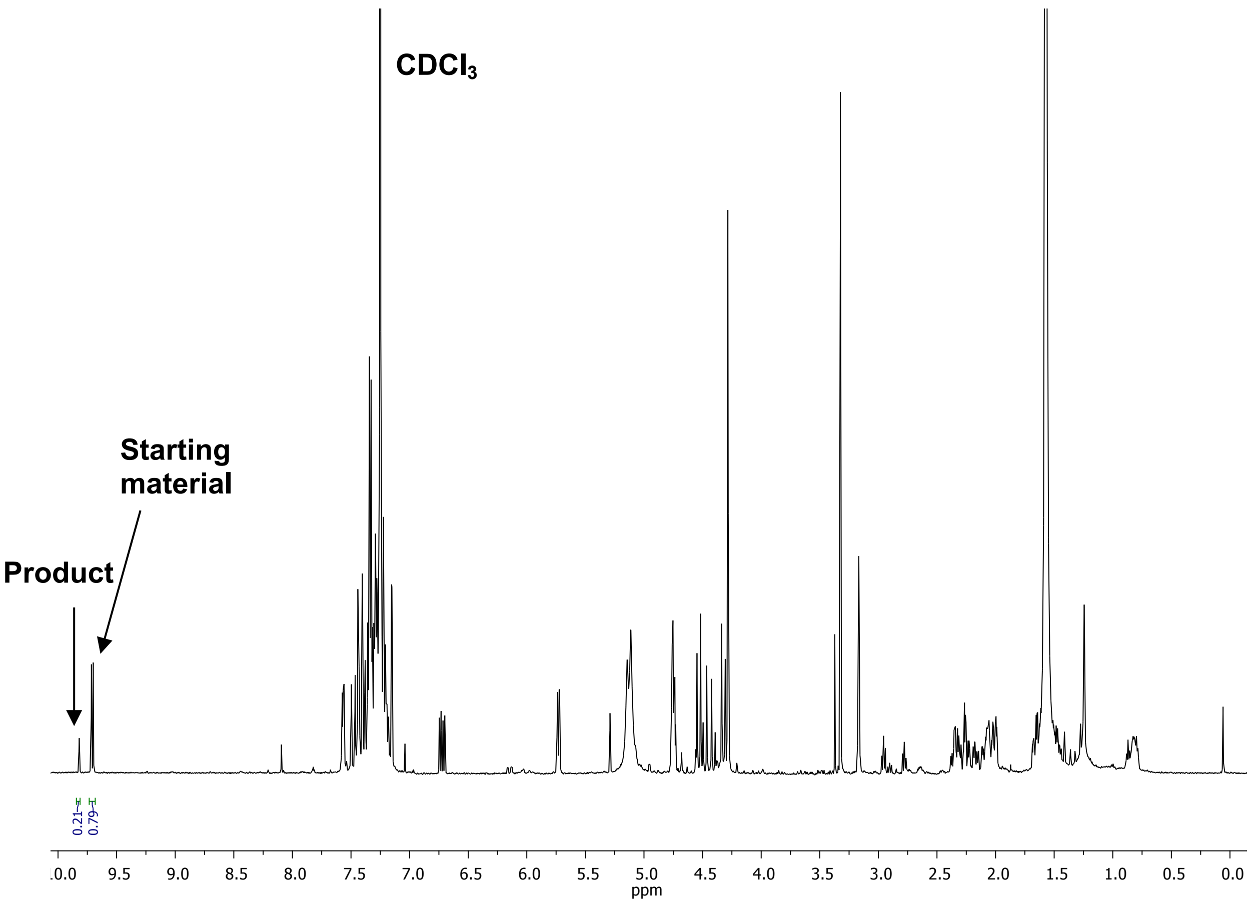

CDCl3
Starting material
Product
0.21
0.79
10.0 9.5 9.0 8.5 8.0 7.5 7.0 6.5 6.0 5.5 5.0 4.5 4.0 3.5 3.0 2.5 2.0 1.5 1.0 0.5 0.0
ppm

6.1.9 M-Sav:2

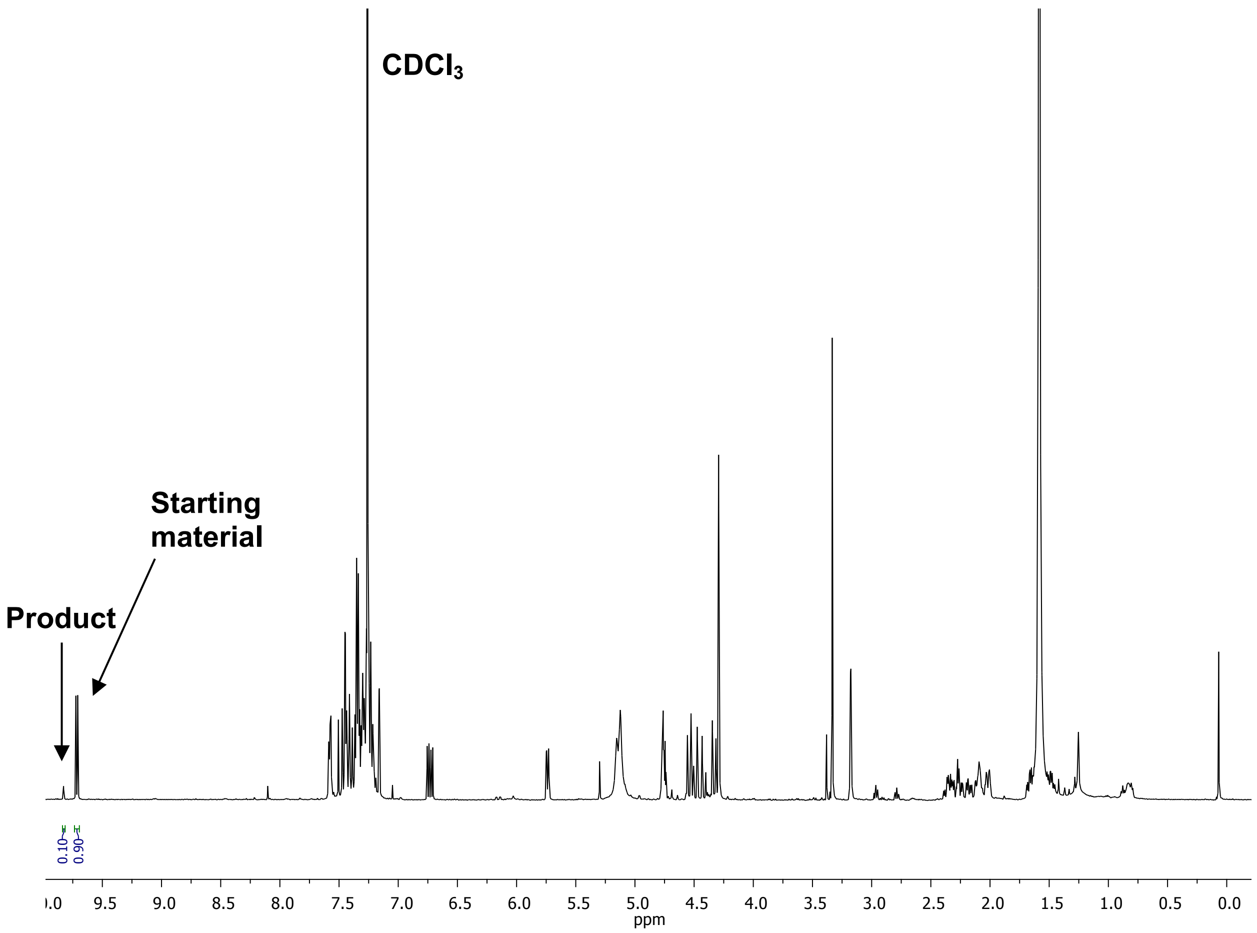

CDCl$_3$
Starting material
Product
0.10
0.90
10.0 9.5 9.0 8.5 8.0 7.5 7.0 6.5 6.0 5.5 5.0 4.5 4.0 3.5 3.0 2.5 2.0 1.5 1.0 0.5 0.0
ppm

6.1.10 T-Sav:1, no shaking (0 rpm)

Formation of an unidentified side-product was observed around 9.25 ppm. The peak around 9.25 ppm resemble the characteristic doublet of an aldehyde, thus the peak was assumed to refer to a single H. Therefore, the estimated conversion of this reaction was made by a ratio between the aldehydic peaks of the starting material **3**, product **13** and this side-product.

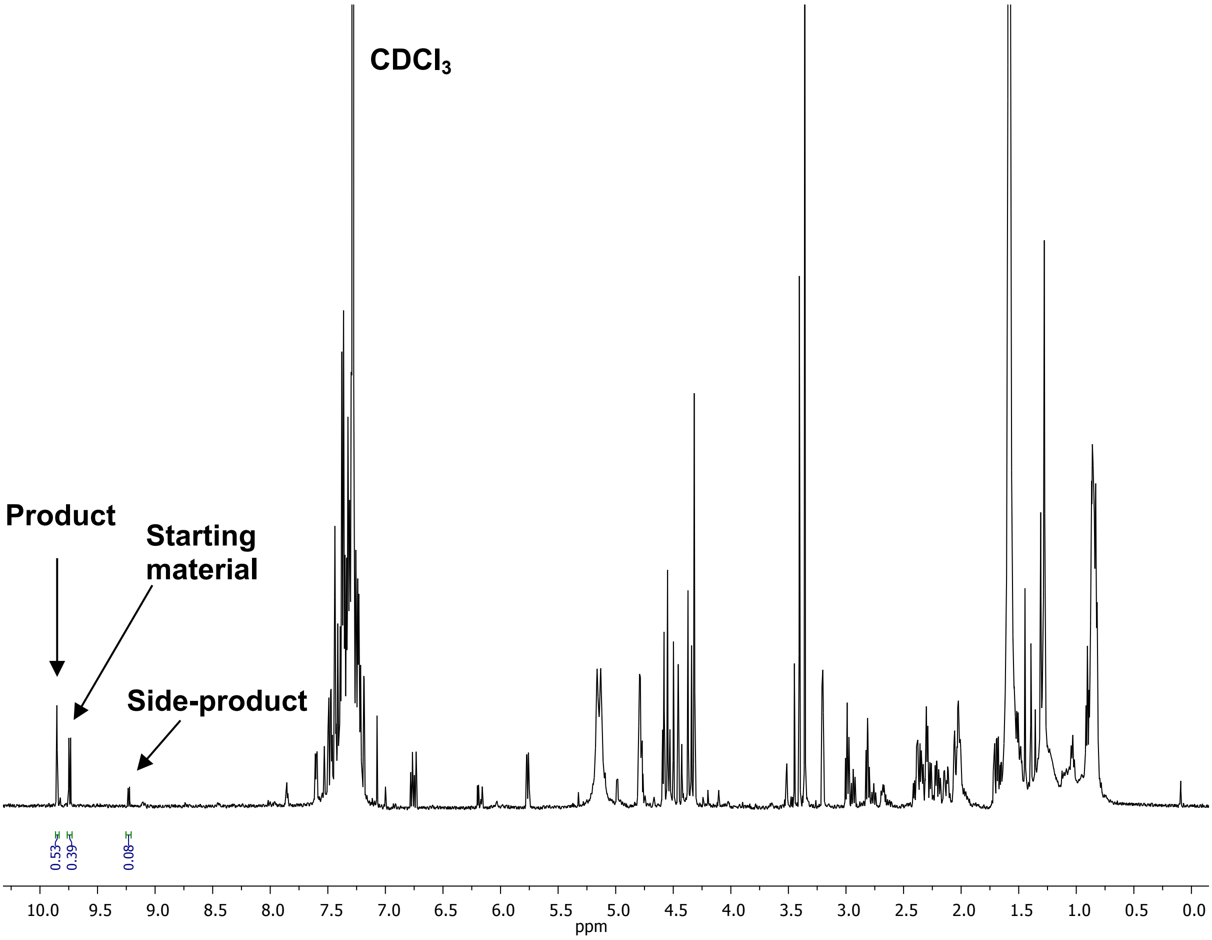

6.1.11 T-Sav:1 (5 equivalents BNAH, 25% MeOH)

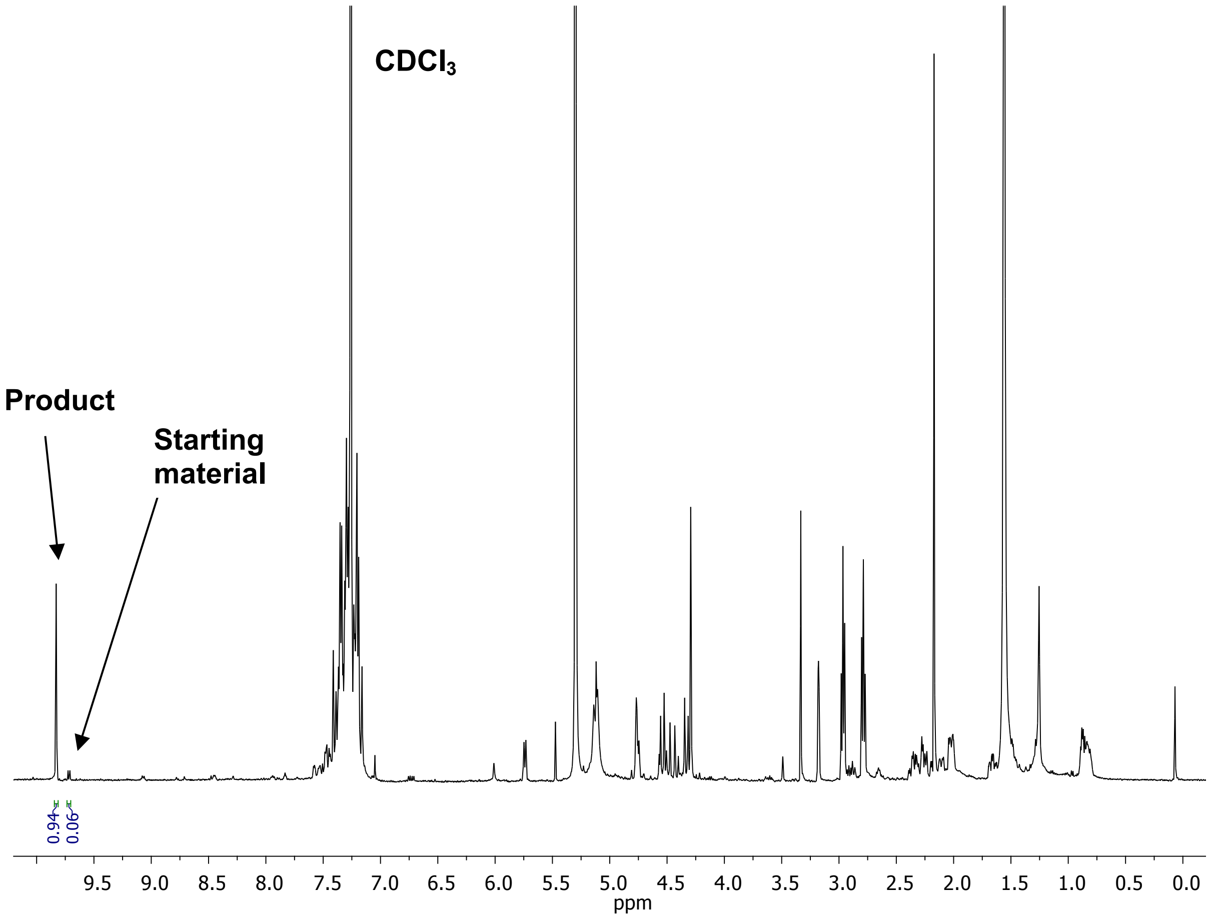

6.1.12 T-Sav:1, 2 mol%

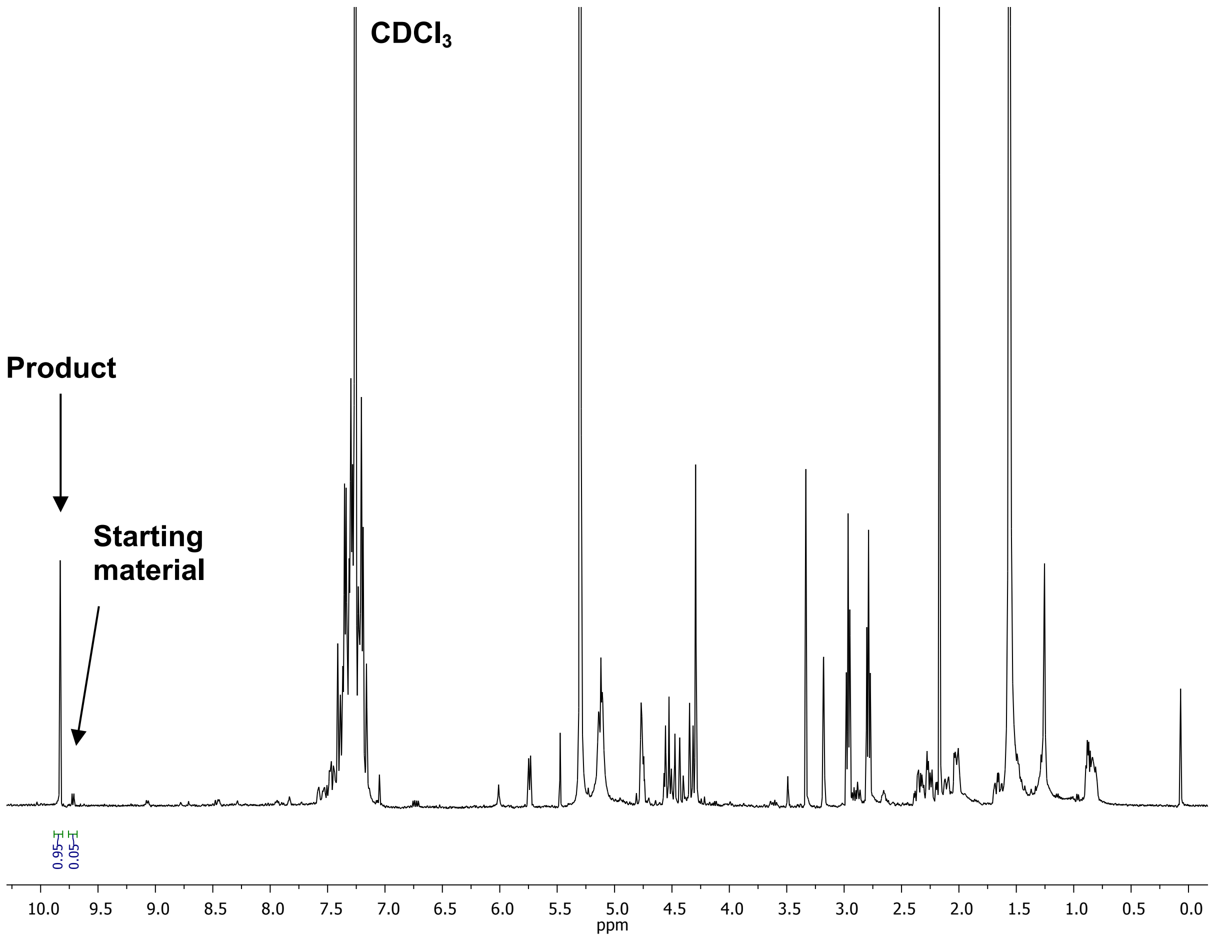

6.2 Hydride Donors Screening Reactions for the Hydride Transfer to Cinnamaldehyde

6.2.1 NADH

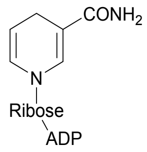

6

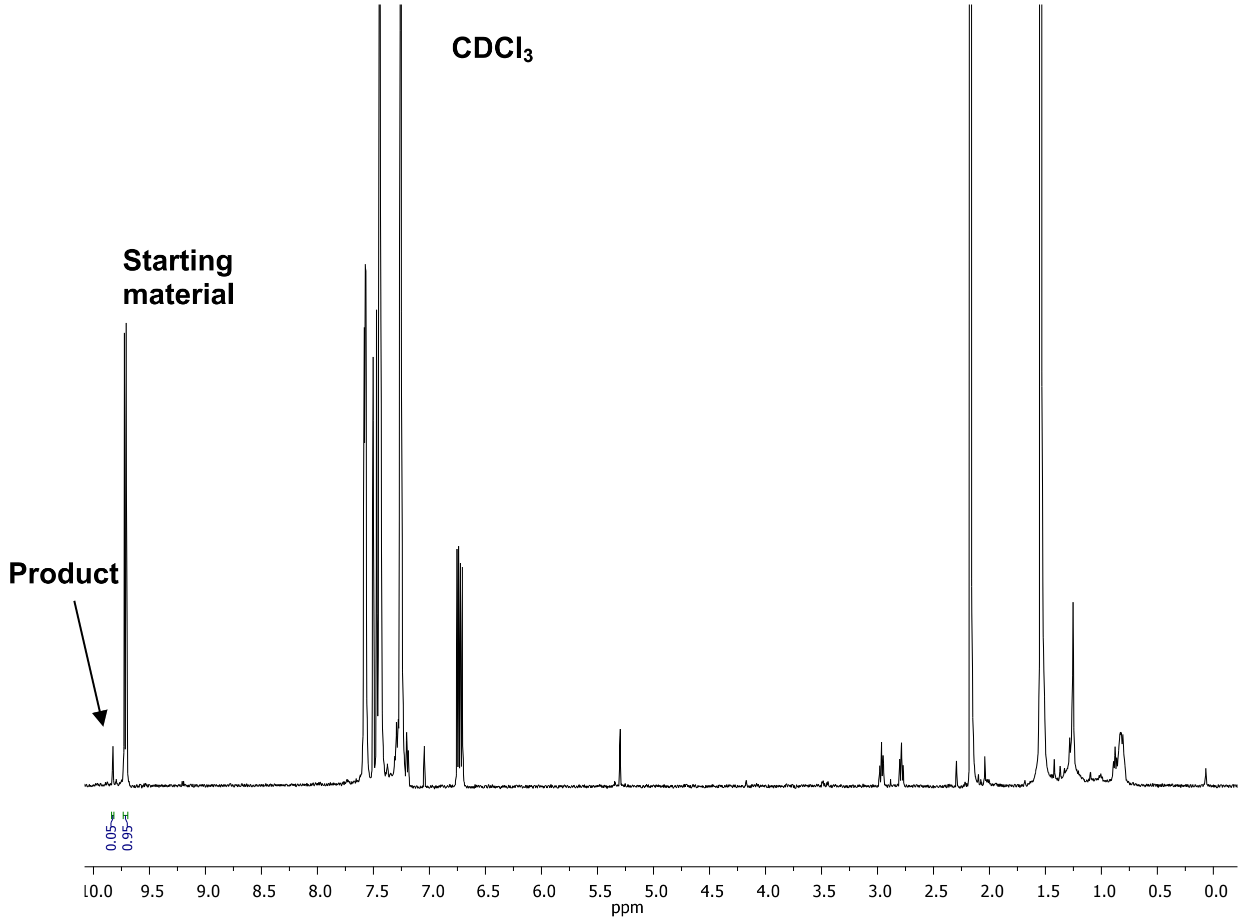

6.2.2 Hantzsch ester

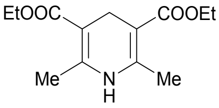

7

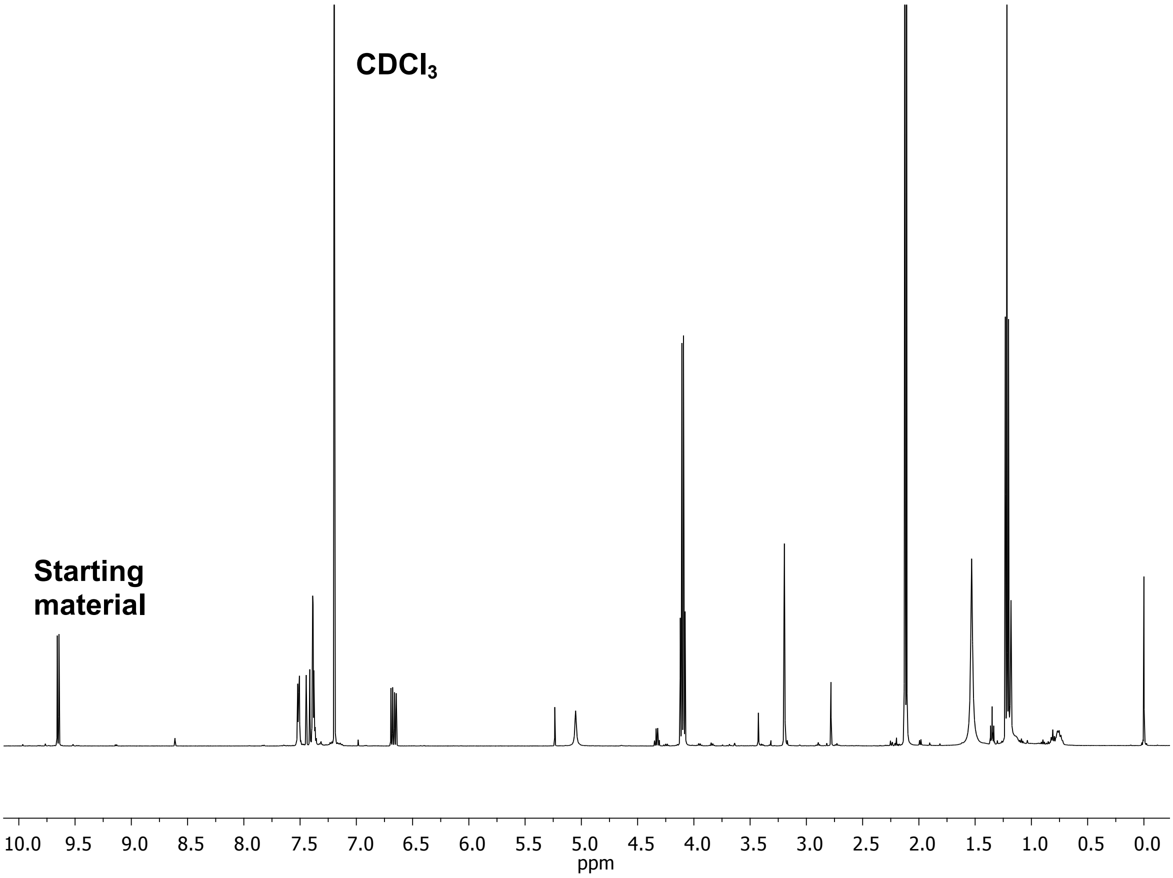

6.3 Substrate Scope for the Hydride Transfer from BNAH to Different A, B-Unsaturated Aldehydes (5 Equivalent BNAH, 25% Methanol as Co-Solvent)

6.3.1 (*E*)-3-(*p*-chlorophenyl) acrylaldehyde

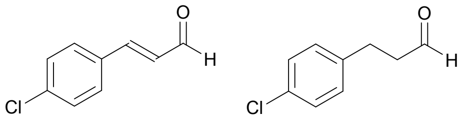

The estimated conversion of (*E*)-3-(4-chlorophenyl) acrylaldehyde (**8**) into 3-(4-chlorophenyl) propanal (**14**) is calculated after integration of the product peak at 9.81 ppm with the substrate peak at 9.63 ppm. The spectroscopic data of **14** are the following: ^{1}**H NMR** (500 MHz, $CDCl_3$): δ = 9.81 (t, *J*= 1.3 Hz, 1 H), 7.28-7.23 (m, 2H), 7.15-7.10 (m, 2H), 2.92 (t, *J*= 7.5 Hz, 2 H), 2.79-2.74 (t, *J*= 7.5 Hz, 2 H) ppm. These data matched those reported in the literature.[7]

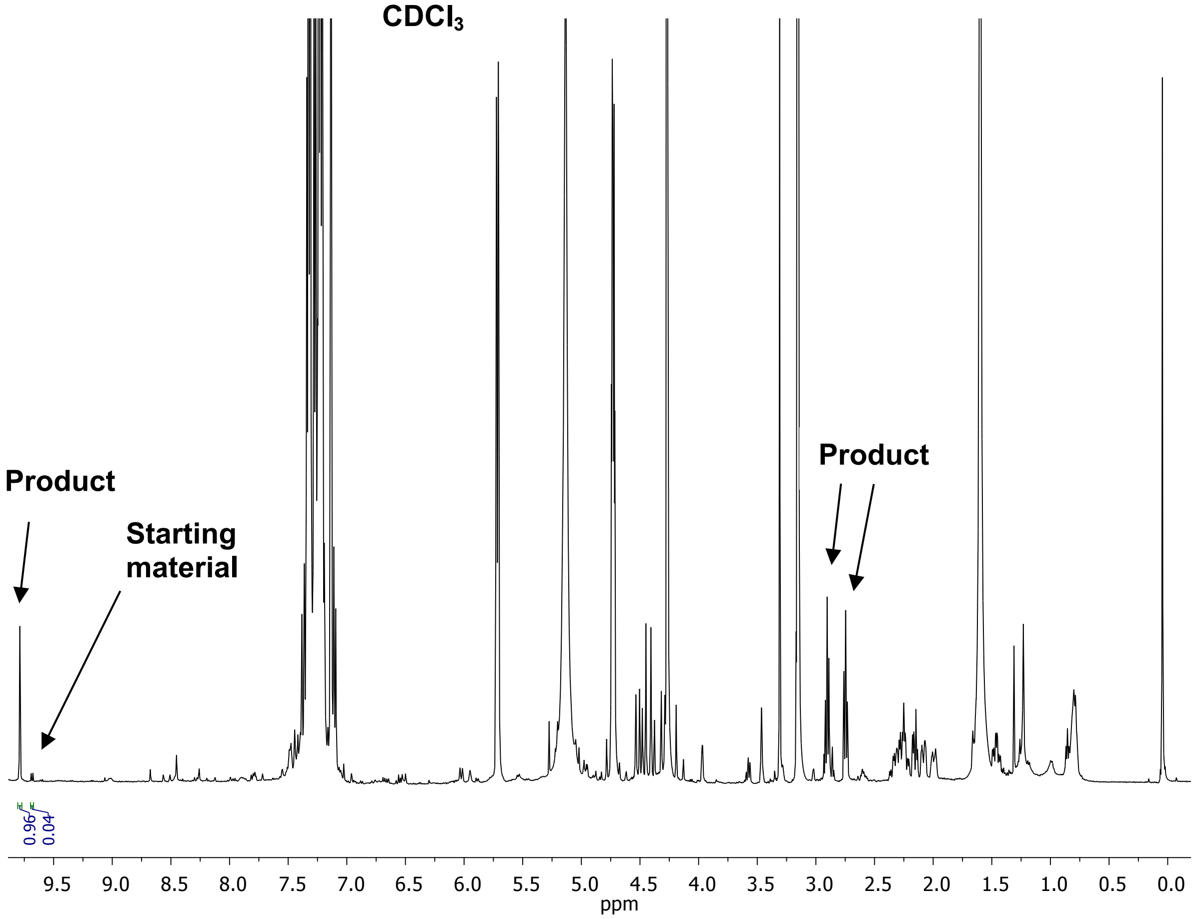

6.3.2 (*E*)-3-(*p*-fluorophenyl) acrylaldehyde

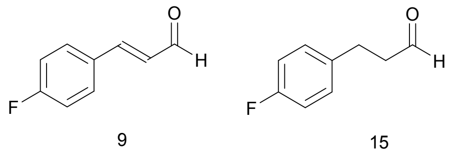

The estimated conversion of (*E*)-3-(4-fluorophenyl) acrylaldehyde (**9**) into 3-(4-fluorophenyl) propanal (**15**) is calculated after integration of the product peak at 9.80 ppm with the substrate peak at 9.63 ppm. The spectroscopic data of **15** are the following: **^{1}H NMR** (500 MHz, $CDCl_3$): δ = 9.80 (t, *J*= 1.3 Hz, 1 H), 7.17-7.11 (m, 2H), 7.00-6.93 (m, 2H), 2.92 (t, *J*= 7.5 Hz, 2 H), 2.76 (t, *J*= 7.5 Hz, 2 H) ppm. These data matched those reported in the literature.[8]

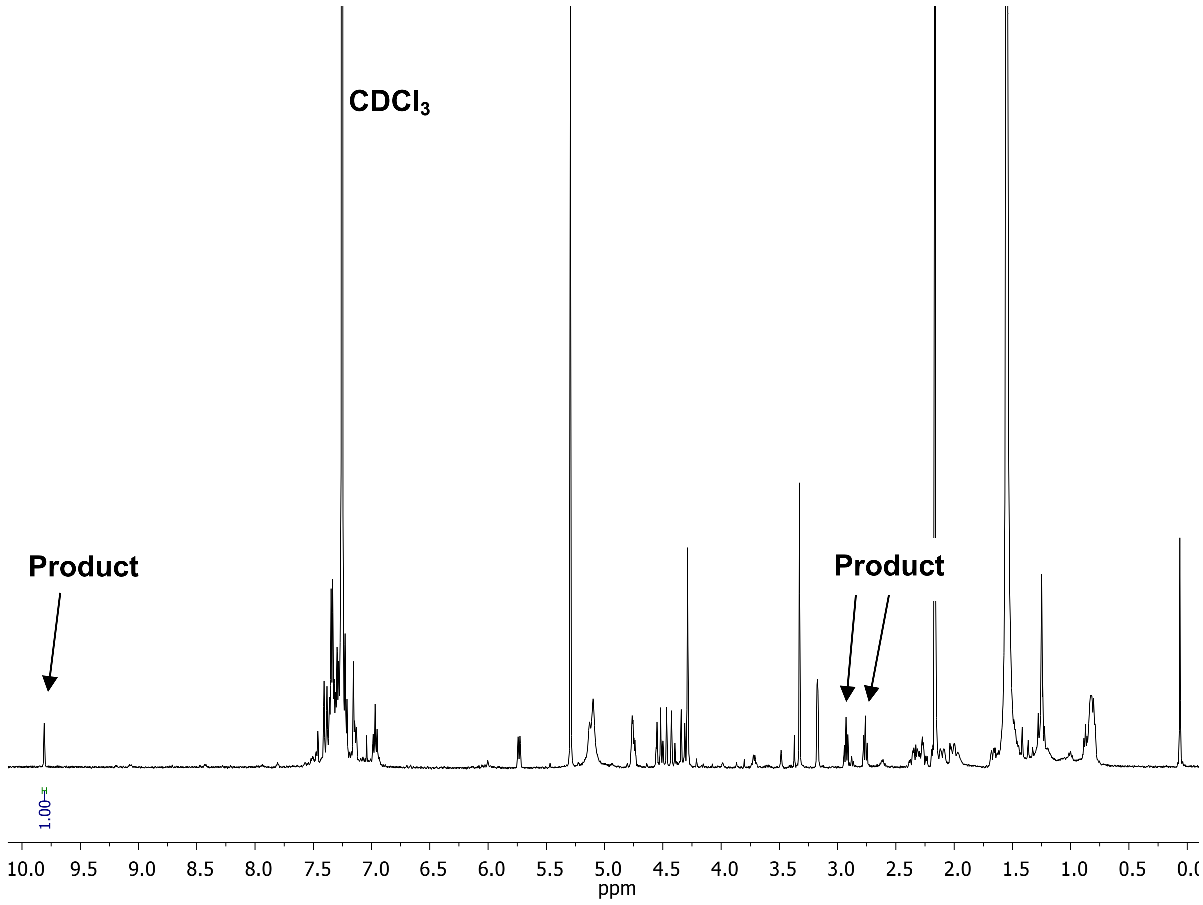

6.3.3 (*E*)-3-(*p*-bromophenyl) acrylaldehyde

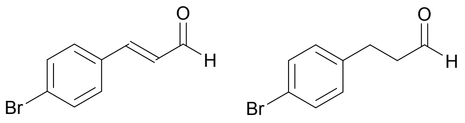

10 16

The estimated conversion of (*E*)-3-(4-bromophenyl) acrylaldehyde (**10**) into 3-(4-bromophenyl) propanal (**16**) is calculated after integration of the product peak at 9.81 ppm with the substrate peak at 9.65 ppm. The spectroscopic data of **16** are the following: **^{1}H NMR** (500 MHz, $CDCl_3$): δ = 9.81 (t, *J*= 1.5 Hz, 1 H), 7.41 (m, 2H), 7.07 (m, 2H), 2.91 (t, *J*= 7.4 Hz, 2 H), 2.77 (t, *J*= 7.3 Hz, 2 H) ppm. These data matched those reported in the literature.[9]

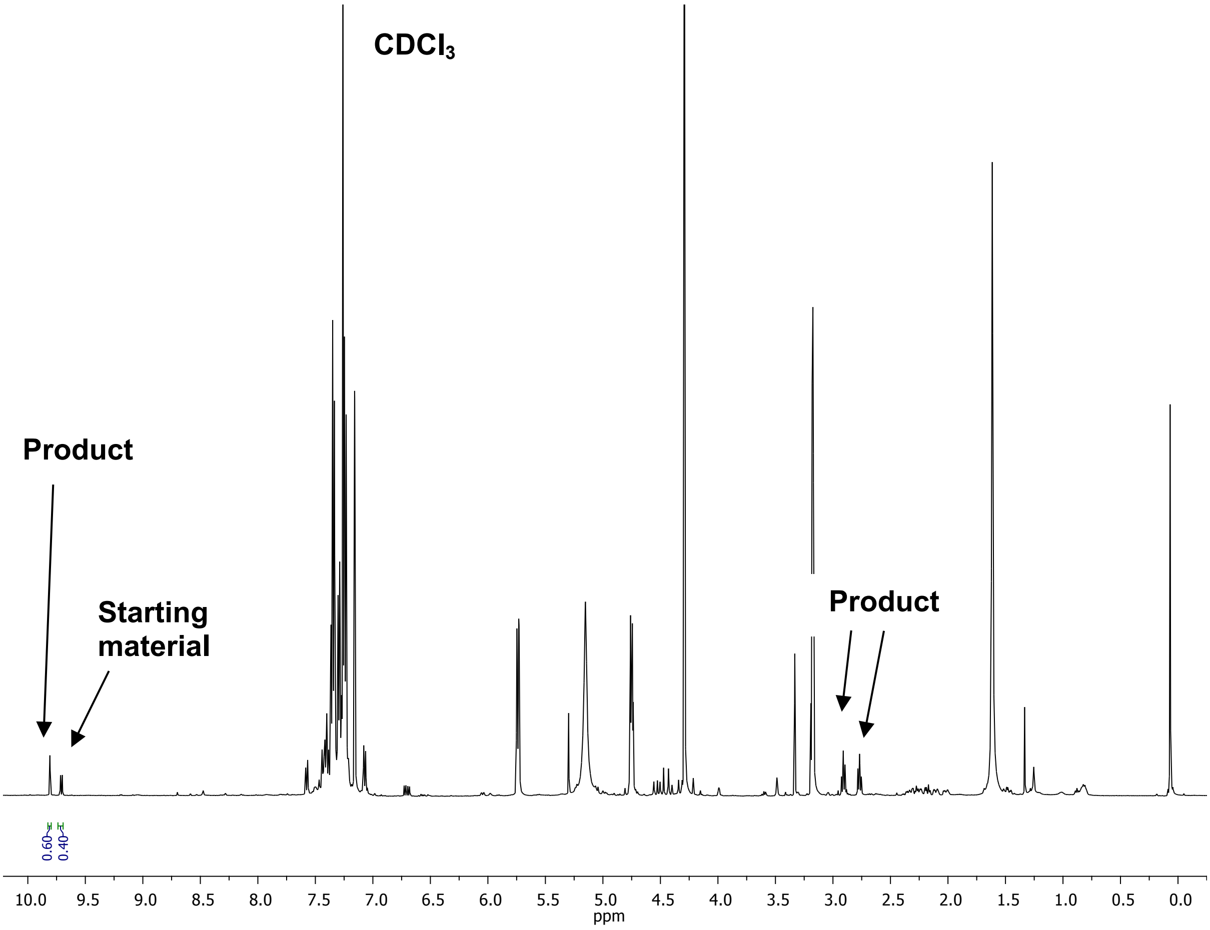

6.3.4 (*E*)-3-(*p*-methoxyphenyl) acrylaldehyde

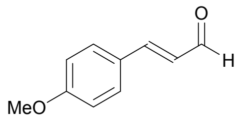

11

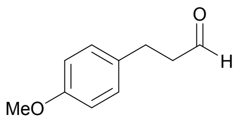

17

The estimated conversion of (*E*)-3-(4-methoxyphenyl) acrylaldehyde (**11**) into 3-(4-methoxyphenyl) propanal (**17**) is calculated after integration of the product peak at 9.84 ppm with the substrate peak at 9.68 ppm. The spectroscopic data of **17** are the following: **^{1}H NMR** (500 MHz, $CDCl_3$): δ = 9.81 (t, *J*= 1.5 Hz, 1 H), 7.14-7.09 (m, 2H), 6.86-6.81 (m, 2H), 3.79 (s, 3H), 2.91 (t, *J*= 7.5 Hz, 2 H), 2.78-2.72 (t, *J*= 7.5 Hz, 2 H) ppm. These data matched those reported in the literature. [7, 8]

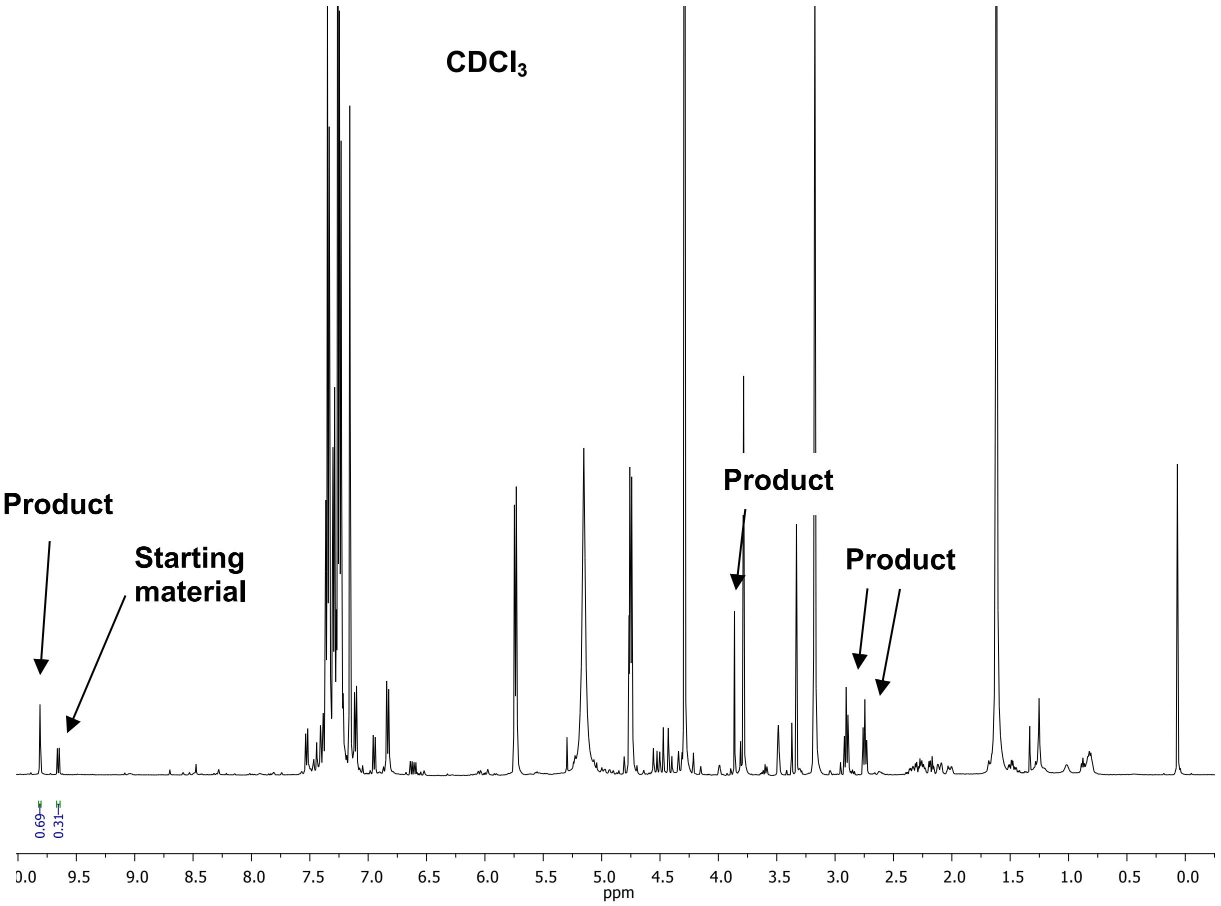

6.3.5 (*E*)-3-(*p*-nitrophenyl) acrylaldehyde

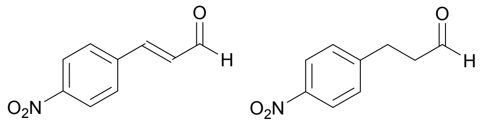

12 18

The estimated conversion of (*E*)-3-(4-nitrophenyl) acrylaldehyde (**12**) into 3-(4-nitrophenyl) propanal (**18**) is calculated after integration of the product peak at 9.84 ppm with the substrate peak at 9.81 ppm. The spectroscopic data of **18** are the following: **^{1}H NMR** (500 MHz, $CDCl_3$): δ = 9.84 (t, *J*= 1.5 Hz, 1 H), 8.15 (m, 2H), 7.37 (m, 2H), 3.07 (t, *J*= 7.5 Hz, 2 H), 2.87 (t, *J*= 7.5 Hz, 2 H), 2.78-2.72 (t, *J*= 7.5 Hz, 2 H) ppm. These data matched those reported in the literature.[10]

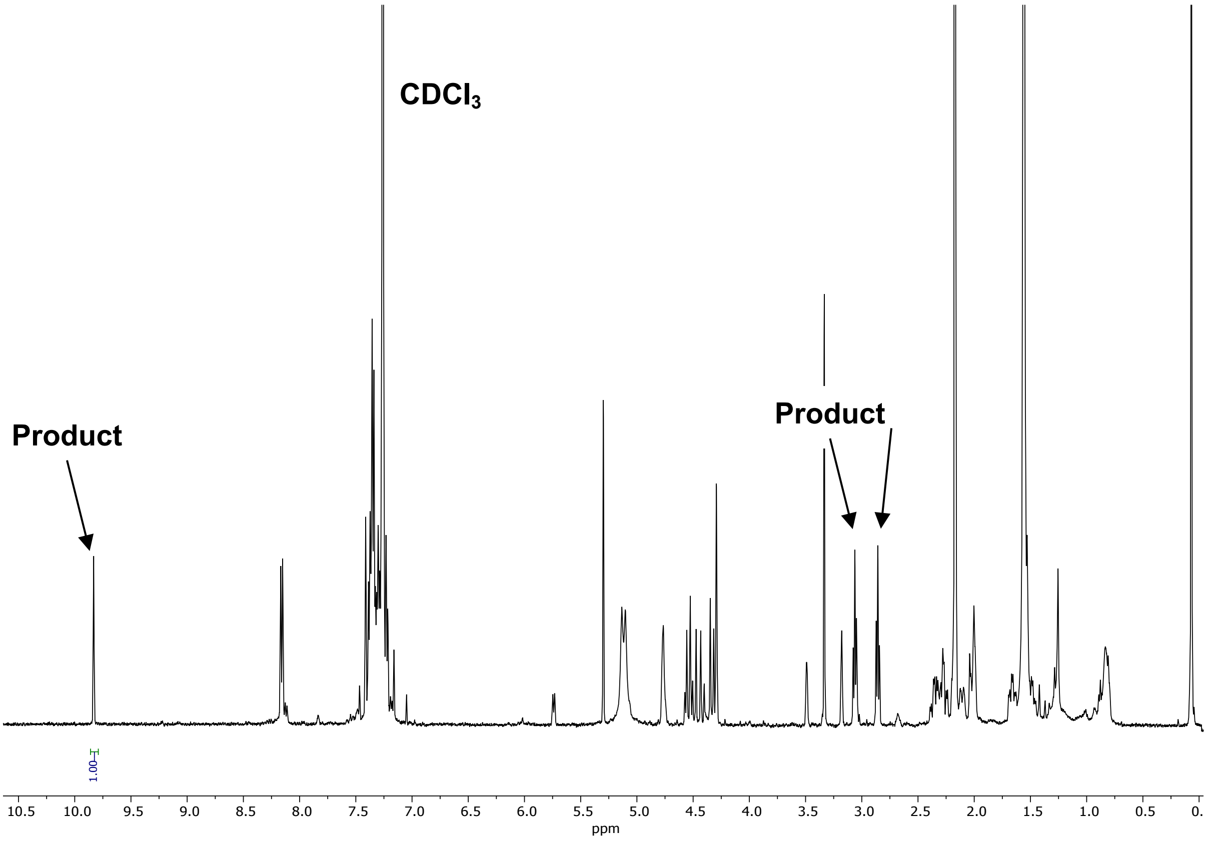

6.3.6 (*E*)-3-(*p*-tolyl) acrylaldehyde

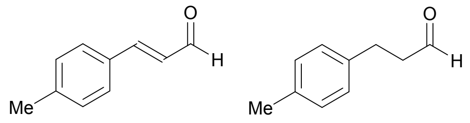

13 19

The estimated conversion of (*E*)-3-(*p*-tolyl) acrylaldehyde (**13**) into 3-(*p*-tolyl) propanal (**19**) is calculated after integration of the product peak at 9.81 ppm with the substrate peak at 9.81 ppm. The spectroscopic data of **19** are the following: **^{1}H NMR** (500 MHz, $CDCl_3$): δ = 9.81 (t, *J*= 1.5 Hz, 1 H), 7.09 (m, 4H), 2.86 (t, *J*= 7.5 Hz, 2 H), 2.69 (t, *J*= 7.5 Hz, 2 H), 2.31 (s, 3H) ppm. These data matched those reported in the literature.[10, 11]

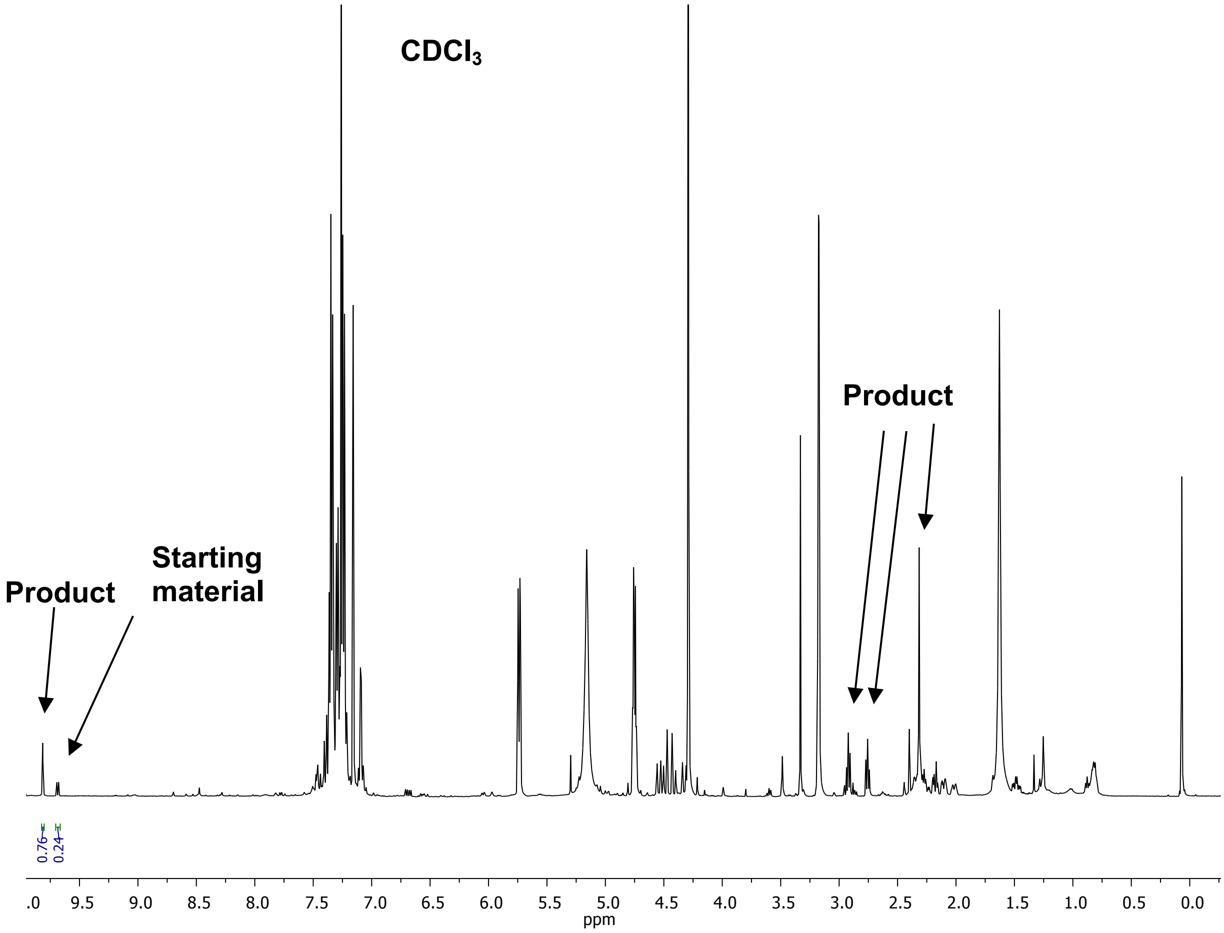

7 Michaelis-Menten Kinetic for the Reaction Between 3 and 4

7.1 Procedure for the Kinetic Assessment

The pseudo-first order kinetic assessment for the transfer hydrogenation of BNAH (**4**) to cinnamaldehyde (**3**) was setup following the procedure reported in the section 3.1 of this supplementary information. Reaction were run in triplicate and stopped after 24 hours, and the estimated conversion was assessed by ^{1}H NMR spectroscopy. Cinnamaldehyde was used at the following concentrations: 0, 0.8, 1.6, 3.3, 4.4, 5.5, 6.6, 7.7 and 8.8 mM. BNAH concentration was maintained constant at 20 mM, meanwhile 0.076 mM of **T-Sav**:**1** complex were employed (**Fig. S3** and **Table S1**).

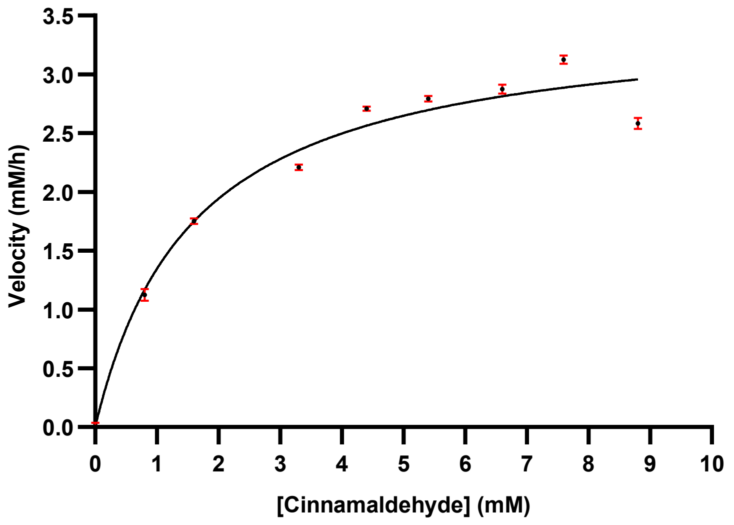

Fig. S3. Kinetic evaluation of **T-Sav**:**1** complex for transfer hydrogenation from BNAH to cinnamaldehyde.

Table S1. Primary KIE observed for different catalytic system for the transfer hydrogenation from BNAH to cinnamaldehyde.

V_{max}	k_{cat}	K_M	k_{cat} /K_M
3.5 ± 0.5 mM·h^{-1}	45.9 ± 0.3 h^{-1}	1.6 ± 0.7 mM	30.6 ± 0.5 mM^{-1} h^{-1} (8.5 M^{-1}·s^{-1})

8 Computational methods.

8.1 Computational details of the molecular models set up

The origin of atom coordinates of streptavidin with bound biotin was adapted from X-Ray structure as available in Protein Data Bank (PDB ID 1STP).[14] Biological assembly, missing atoms, protonation state of titratable amino acids, optimization and molecular dynamics (MD) simulations performed for the **T-Sav** with biotin-catalyst were described in our previous paper.[1]

In brief, after adding missing hydrogen atoms, the system was neutralized by addition of 7 sodium counter ions and placed in the box of 87 ∅ 86 ∅ 76 Å^3 of TIP3P[15] water molecules. Then, optimization and molecular dynamics (MD) simulations were performed at molecular mechanistic (MM) level using NAMD software,[16] with the AMBER ff-03 parameters.[17] Missing atoms types, charges and parameters were generated using the Antechamber software package together with a general AMBER force field (GAFF)[18, 19] available in Amber Tools. NPT MD of 10 ns with time step of 1 fs at 300 K were carried out after previous optimization, heating (from 0 to 300 K with 0.001 K temperature increment) and equilibration processes of 100ps. The constant temperature and pressure were controlled using the Langevin piston method.[20] Periodic boundary conditions (PBC) using the particle mesh Ewald method were applied. A Cut-off for nonbonding interactions was applied using a smooth switching function between 14.5 to 16 Å. Analysis of the time evolution of the RMSD of the backbone atoms confirmed that the system was equilibrated after series of MD equilibration, consisting in 3 steps: 1) 10ns of MD with Sav-biotin complex; 2) 4 ns MD of biotin linked with the substrate; and 3) 20ns of MD of biotin with substrate and BNAH (**Fig. S4**). Last structure from 10 ns MDs was used in order to explore the hydride transfer step from BNAH to cinnamaldehyde in the active site of T-Sav. After optimization of the system, those residues located 20 Å beyond any atoms of the substrate were kept frozen in the remaining calculations. As explained below, potential energy surfaces, free energy surfaces and spline corrections have been performed using fDynamo[21] library together with implemented AMBER force field.[22]

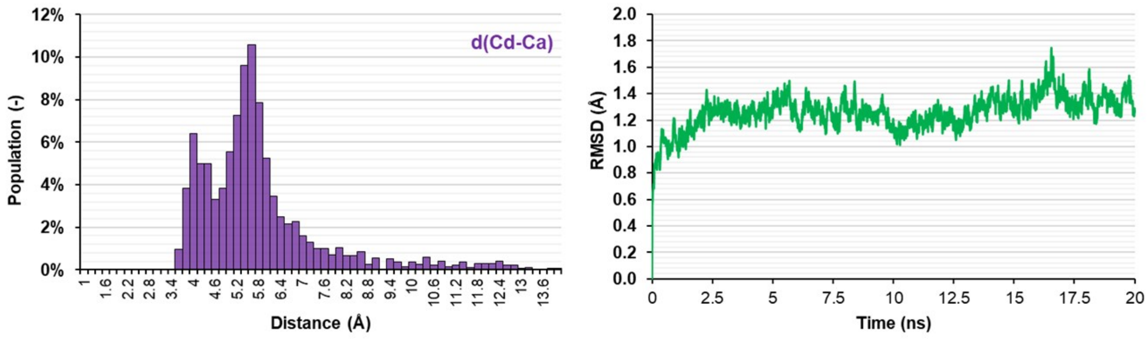

Fig. S4. Population analysis of structures along the 20 ns of MD simulation as a function of the hydride donor-acceptor distance. Time evolution of the RMSD of the backbone atoms of T-Sav.

Table S2. Force field Parameters for BNAH obtained from Antechamber software used in classical MD simulations.

BNAH Structure

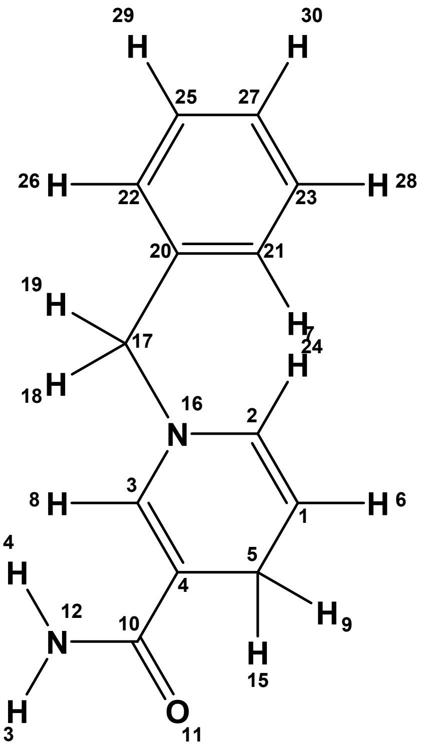

Atom Atom Type Charge

Atom	Atom Type	Charge
C1	c2	-0.221200
C2	c2	0.023000
C3	c2	0.100000
C4	ce	-0.257400
C5	c3	0.027000
H6	ha	0.138000
H7	h4	0.143000
H8	h4	0.147000
H9	hc	0.047700
C10	c	0.678300
O11	o	-0.611100
N12	n	-0.677000
H13	hn	0.299500
H14	hn	0.310500
H15	hc	0.069700
N16	nh	-0.536200
C17	c3	0.258100
H18	h1	0.060700
H19	h1	0.068700
C20	ca	-0.127300
C21	ca	-0.121000
C22	ca	-0.114000
C23	ca	-0.130000
H24	ha	0.131000
C25	ca	-0.127000
H26	ha	0.138000
C27	ca	-0.123000
H28	ha	0.134000
H29	ha	0.136000
H30	ha	0.134000

NONBON

c2	1.9080	0.0860
h4	1.4090	0.0150
ha	1.4590	0.0150
c3	1.9080	0.1094
hc	1.4870	0.0157
ce	1.9080	0.0860
c	1.9080	0.0860
o	1.6612	0.2100
n	1.8240	0.1700
hn	0.6000	0.0157
nh	1.8240	0.1700
h1	1.3870	0.0157
ca	1.9080	0.0860

BOND

c2-h4	348.60	1.084
c2-c2	589.70	1.324
c2-nh	462.60	1.355
c2-ha	344.30	1.087

ANGLE

c2-c2-ha	50.040	120.940
c2-c2-c3	64.330	123.420
c2-nh-c2	65.540	124.500
c2-nh-c3	63.170	123.710
h4-c2-c2	49.750	122.540
h4-c2-nh	51.550	115.640
c2-c2-nh	69.800	124.990
c2-c3-hc	47.030	110.490
c2-c3-ce	64.080	111.750
ha-c2-c3	45.660	117.300
c3-ce-c	63.430	116.630
c3-ce-c2	64.300	122.890
hc-c3-hc	39.430	108.350
hc-c3-ce	47.000	110.980
ce-c -o	69.270	122.920
ce-c -n	68.880	115.010
ce-c2-h4	49.380	122.290
ce-c2-nh	70.640	120.720
c -ce-c2	65.820	120.420

c2-c3	328.30	1.508	c -n -hn	49.210	118.460
c3-hc	337.30	1.092	o -c -n	75.830	122.030
c3-ce	331.30	1.505	hn-n -hn	39.730	117.850
ce-c	363.80	1.474	nh-c3-h1	49.730	109.960
ce-c2	560.50	1.339	nh-c3-ca	66.640	111.410
c -o	648.00	1.214	c3-ca-ca	63.840	120.630
c -n	478.20	1.345	h1-c3-h1	39.180	109.550
n -hn	410.20	1.009	h1-c3-ca	46.780	110.950
nh-c3	332.70	1.458	ca-ca-ha	48.460	120.010
c3-h1	335.90	1.093	ca-ca-ca	67.180	119.970
c3-ca	323.50	1.513			
ca-ca	478.40	1.387			
ca-ha	344.30	1.087			

DIHE					
c2-c2-c3-hc	1	0.380	180.000	-3.000	
c2-c2-c3-hc	1	1.150	0.000	1.000	
c2-c2-c3-ce	1	0.000	0.000	2.000	
c2-nh-c2-ce	1	0.675	180.000	2.000	
c2-nh-c2-h4	1	0.675	180.000	2.000	
c2-nh-c3-h1	1	0.000	0.000	2.000	
c2-nh-c3-ca	1	0.000	0.000	2.000	
h4-c2-c2-ha	1	6.650	180.000	2.000	
h4-c2-c2-c3	1	6.650	180.000	2.000	
h4-c2-nh-c3	1	0.675	180.000	2.000	
c2-c2-nh-c2	1	0.675	180.000	2.000	
c2-c2-nh-c3	1	0.675	180.000	2.000	
c2-c3-ce-c	1	0.000	0.000	2.000	same as X - c2-c3-X
c2-c3-ce-c2	1	0.000	0.000	2.000	same as X - c2-c3-X
ha-c2-c2-nh	1	6.650	180.000	2.000	
ha-c2-c3-hc	1	0.000	0.000	2.000	
ha-c2-c3-ce	1	0.000	0.000	2.000	
c3-c2-c2-nh	1	6.650	180.000	2.000	
c3-ce-c -o	1	2.175	180.000	2.000	
c3-ce-c -n	1	2.175	180.000	2.000	
c3-ce-c2-h4	1	6.650	180.000	2.000	
c3-ce-c2-nh	1	6.650	180.000	2.000	
hc-c3-ce-c	1	0.000	0.000	2.000	same as X - c2-c3-X
hc-c3-ce-c2	1	0.380	180.000	-3.000	same as hc- c3-c2-c2
hc-c3-ce-c2	1	1.150	0.000	1.000	same as hc- c3-c2-c2
ce-c -n -hn	1	2.500	180.000	2.000	
ce-c2-nh-c3	1	0.675	180.000	2.000	
c -ce-c2-h4	1	6.650	180.000	2.000	
c -ce-c2-nh	1	6.650	180.000	2.000	
o -c -ce-c2	1	2.175	180.000	2.000	
o -c -n -hn	1	2.500	180.000	-2.000	
o -c -n -hn	1	2.000	0.000	1.000	

n -c -ce-c2	1	2.175	180.000	2.000
nh-c3-ca-ca	1	0.000	0.000	2.000
c3-ca-ca-ha	1	3.625	180.000	2.000
c3-ca-ca-ca	1	3.625	180.000	2.000
h1-c3-ca-ca	1	0.000	0.000	2.000
ca-ca-ca-ha	1	3.625	180.000	2.000
ca-ca-ca-ca	1	3.625	180.000	2.000
ha-ca-ca-ha	1	3.625	180.000	2.000

8.2 Computational details of the QM/MM simulations

An additive hybrid Quantum Mechanics/Molecular Mechanics (QM/MM) scheme was employed for the construction of the total Hamiltonian, $\hat{H}_{eff}$, where the total energy is obtained from the sum of each contribution to the energy.

$$\hat{H}_{eff} = \hat{H}_{QM} + \hat{H}_{QM/MM}^{elec} + \hat{H}_{QM/MM}^{VdW} + \hat{H}_{MM} \quad \text{(S1)}$$

Here, $\hat{H}_{QM}$ describes the atoms in the QM part, $\hat{H}_{QM/MM}$ defines the interaction between the QM and MM region and $\hat{H}_{MM}$ describes the rest of the MM part. As shown in **Scheme S2**, the QM subset of atoms includes part of the biotin catalyst, the cinamaldehyde, the nitromethane, a molecule of water and part of Lys_{121D}.

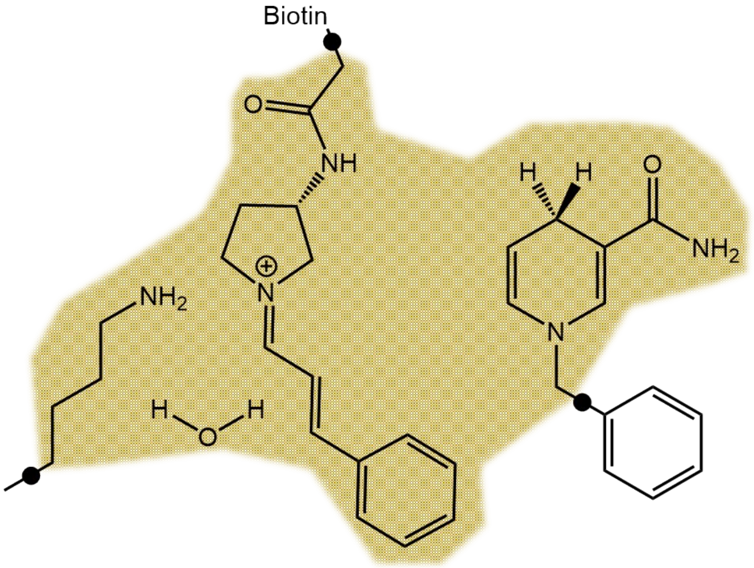

Scheme S2. Schematic representation of the active site of **T-Sav**. QM region is represented as orange shaded region. Black dots represent the introduced quantum link atoms between the QM and the MM regions.

8.3 Potential Energy Surfaces

Exploration of the Potential Energy Surfaces (PES) was carried out by choosing the appropriate combination of internal coordinates (ξ_i). Thus, a first attempt to explore a two-dimension PES explicitly controlling the hydride transfer and the distances defining the rehybridization of the carbon atoms, confirms that the two processes are not coupled: first the internal electron transfer takes place, followed by the hydride transfer (**Fig. S5**). Then, the antisymmetric combination of distances defining the position of the transferring hydride from

the donor to the acceptor atoms was used as reaction coordinate. A harmonic constraint was used to maintain the proper interatomic distances along the reaction coordinate, and a series of conjugate gradient optimizations and L-BFGS-B optimization algorithms were applied to obtain the final potential energy of the minimized constrained geometry. The QM sub-set of atoms were described first by the RM1 semiempirical Hamiltonian[23] and later by the M06-2X functional with the standard 6-31+G(d,p) basis set using the Gaussian09 program.[24]

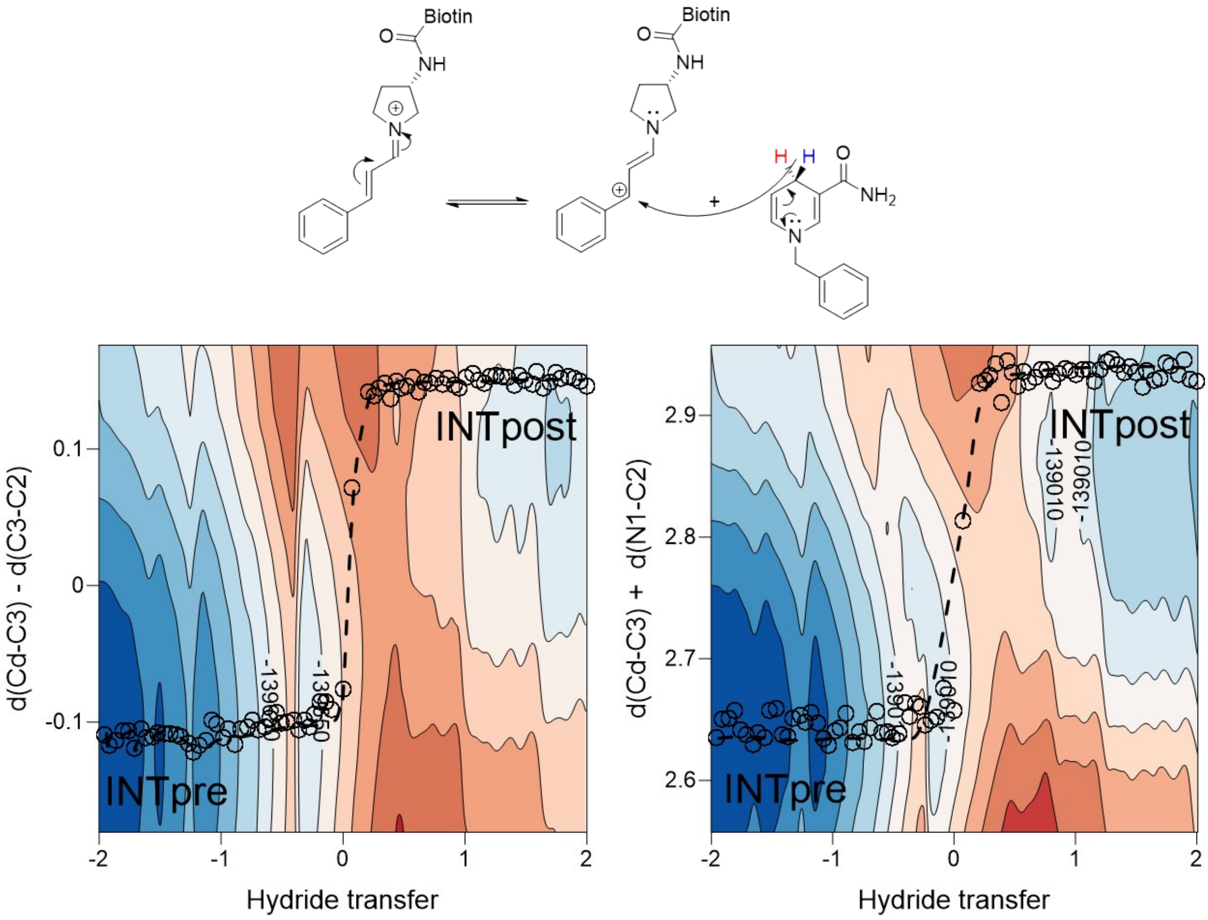

Fig. S5. RM1/MM PES computed for the hydride transfer step. Y axis represents the antisymmetric combination of distances escribing the rehybridization of the acceptor moiety while X axis represents the antisymmetric combination of distances escribing the hydride transfer (Cd-H) – (H-Ca). Circles and dashed line represent the structures along the IRC traced down from the hydride transfer transition state. Distances in Å.

8.4 Free Energy Surfaces

FESs were obtained, in terms of a Potential of Mean Force (1D-PMF), using the Umbrella Sampling approach[25] combined with the Weighted Histogram Analysis Method (WHAM).[26] Series of MD simulations were performed adding a constraint along the selected reaction coordinates with an umbrella force constant of 2500 $kJ \cdot mol^{-1} \cdot Å^{-2}$. In every window QM/MM MD simulations were performed with a total of 5 ps of equilibration and 20 ps of production at 303 K using the Langevin-Verlet algorithm[27] with a time step of 1 fs. Structures obtained in previously computed PESs were used as starting points for the MD simulations in every window.

8.5 Spline Corrections

A correction term is interpolated to any value along the reaction coordinates, ξ, in the FES. A continuous energy function is used to obtain the corrected PMFs:

$$E = E_{LL/MM} + S[\Delta E_{LL}^{HL}(\xi)] \quad \text{(S2)}$$

where S is the spline function and ΔE_{LL}^{HL} is the difference between the energies obtained at low-level (LL) and high-level (HL) of theory of the QM part. The RM1 semiempirical Hamiltonian was used as LL method, while a density functional theory (DFT)-based method was selected for the HL energy calculation. In particular, HL energy calculations were performed by means of the hybrid M06-2X functional[28] using the standard 6-31+G(d,p) basis set. These calculations were carried out using the Gaussian09 program. The resulting Free Energy Surface is shown in **Fig. S6** while evolution of key-interatomic distances and dihedral angle along the PMF of the hydride transfer step computed at M06-2X:RM1/MM level is shown in **Fig. S7**.

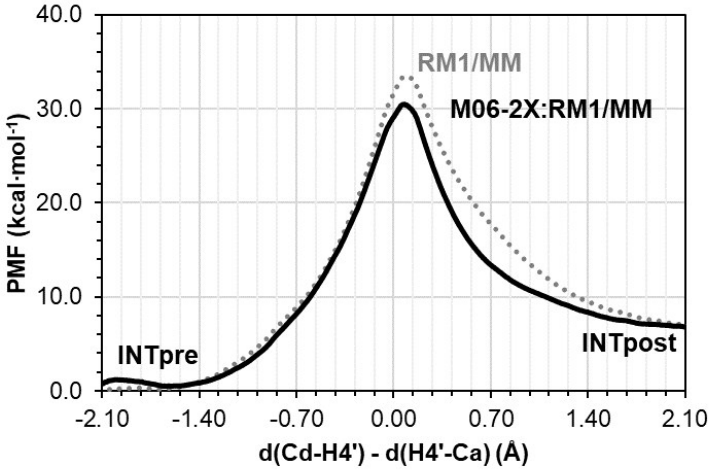

Fig. S6. RM1/MM and M06-2X:RM1/MM free energy surfaces for the hydride transfer step.

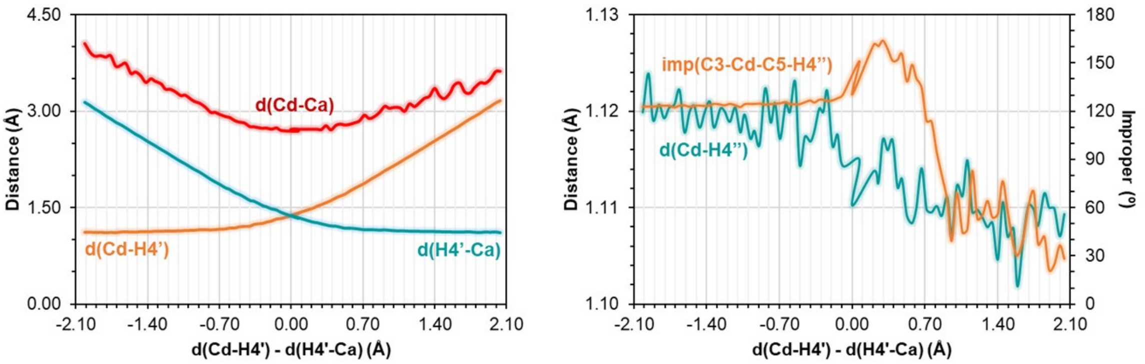

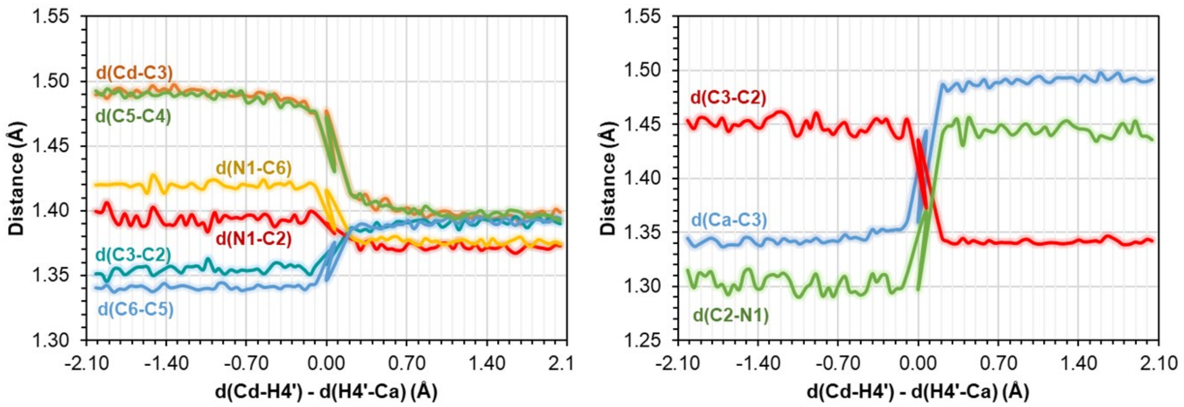

Figure S7. Evolution of key-interatomic distances and dihedral angle along the PMF of the hydride transfer step computed at M06-2X:RM1/MM level.

8.6 Quantum tunnelling corrections

Taking into account that the chemical step under study involves the transfer of a light particle (hydride transfer), the quantum tunelling contribution was evaluated under the framework of Ensemble Averaged Variational Transition State Theory (EA-VTST):[29-33]

$$k(T) = \kappa(T)\frac{k_B T}{h}e^{-\left(\frac{\Delta G_{act}^{QC}(T,\xi)}{RT}\right)} = \frac{k_B T}{h}e^{-\left(\frac{\Delta G_{eff}(T,\xi)}{RT}\right)} \quad \text{(S3)}$$

where R is the ideal gas constant, T is the temperature, k_B is the Boltzmann constant, h is Planck's constant, and ΔG_{eff} is the effective activation free energy, which includes all the contributions to the rate constant, and can be readily compared to the value derived from the experimental rate constant. $\kappa(T)$ is the tunneling coefficient that accounts for reactive trajectories that do not reach the classical threshold energy. ΔG_{act}^{QC} is the quasi-classical activation free energy calculated along the reaction coordinate ξ:[32, 33]

$$\Delta G_{act}^{QC}(T,\xi) = \Delta G_{act}^{CM}(T,\xi) + \Delta G_{vib}^{QM}(T) \quad \text{(S4)}$$

where $\Delta G_{act}^{CM}(T,\xi)$ is the activation free energy obtained from the classical PMF along the selected reaction coordinate, and $\Delta G_{vib}^{QC}(T)$ is the correction term due to the quantized nature of molecular vibrations (mainly zero-point energies).[32, 34-36] In the present work, calculation of the tunneling transmission coefficient, κ(T), were calculated with the small-curvature tunneling (SCT) approximation, which includes reaction-path curvature appropriate for enzymatic hydride transfers.[34, 37, 38] The final tunneling contribution (see main text) is obtained as the average over the reaction paths of 10 TS structures.

8.7 Isotope effects

Quasi-classical equilibrium (BIE and EIE)[39] and kinetic isotope effects (KIEs) have been computed for isotopic substitutions of key atoms from 100and 16 couples of stationary structures, comparing the hydride transfer TSs and reactant complex,pre-transfer and post-

transfer intermediates, Michaelis complex and BNAH in the water solvent at RM1/MM and M06-2X/MM levels of theory, respectively. Then, the ratio between the rate constants corresponding to the light atom “L” (protium) and the heavier isotope “H” (deuterium) can be computed using the Transition State Theory (TST), as:

$$KIE = \frac{(\kappa(T))_L \left(\frac{Q_a}{Q_b}\right)_L}{(\kappa(T))_H \left(\frac{Q_a}{Q_b}\right)_H} e^{-\frac{1}{RT}(\Delta ZPE_L - \Delta ZPE_H)} \quad \text{(S5)}$$

In eq. S5, the subscripts *H* and *L* refers to heavy (deuterium) and light (protium) isotopologs, respectively, the total partition function, *Q*, was computed as the product of the translational, rotational, and vibrational partition functions for the isotopologs in the two stationary structures under comparison, *a* and *b*. ΔZPE refers to the difference in the zero point energies between *a* and *b*. The isotopic effect of the tunneling is considered as the pre-exponential factor $(\kappa(T))_L/(\kappa(T))_H$. The subset of atoms used to define the Hessian for these KIE calculations were those of the QM region, consistent with the “cut-off rule” and the local nature of isotope effects.[40] The Born−Oppenheimer, rigid-rotor, and harmonic oscillator approximations were considered to independently compute the different contributions. Two set of calculations were carried out, considering the ground state as the BNAH species in aqueous solution, or in the active site of the T-Sav.

Table S3. Quasi-classical BNAH/D isotope effects for [1,2-^{2}H] isotopic substitution computed at RM1/MM (10x10 combinations) and M06-2X/MM (4x4 combinations) level. BIE refers to the binding isotope effects; KIE(BNAH^{aq}✂TS) is the KIE considering the equilibrium between the ground state of BNAH in aqueous solution and the hydride transfer transition state; KIE(MC✂TS) is the KIE considering the equilibrium between the Michaelis complex and the hydride transfer transition state; KIE(INT^{pre}✂TS) is the KIE considering the equilibrium between the intermediate previous to the hydride transfer TS, and the hydride transfer transition state; EIE(BNAH^{aq}✂INT^{post}) is the EIE between the ground state of BNAH in aqueous solution and the product state after the hydride transfer transition state; and EIE(MC✂INT^{post}) is the EIE between the Michaelis complex and the product state after the hydride transfer transition state.

Method	BIE	KIE(BNAH^{aq}✂TS)	KIE(MC✂TS)	KIE(INT^{pre}✂TS)	EIE(BNAH^{aq}✂INT^{post})	EIE(MC✂INT^{post})
RM1/MM	0.955 ± 0.017	2.74 ± 0.18	2.87 ± 0.19	2.83 ± 0.18	0.967 ± 0.013	1.013 ± 0.020
M06-2X/MM	0.910 ± 0.030	3.89 ± 0.15	4.28 ± 0.18	4.11 ± 0.15	0.932 ± 0.018	1.025 ± 0.028

Table S4. Tunneling effects on the reaction with the light BNAH (κ^{HH}) and with the double isotopically substituted [1,2-^{2}H] on carbon C4 (κ^{DD}), computed at 298 K, and contribution of tunnelling effects on the KIE (κ^{HH}/κ^{DD}).

	(1)	(2)	(3)	(4)	(5)	(6)	(7)	(8)	(9)	(10)	Average values
κ^{HH}	11.75	11.23	23.36	10.97	5.84	3.33	12.41	11.23	2.23	18.19	11.05 ± 6.08
κ^{DD}	7.92	6.95	15.13	9.84	5.23	3.82	8.34	6.95	2.44	15.36	8.20± 4.08
KIE$^{\kappa}$	1.48	1.61	1.54	1.11	1.12	0.87	1.49	1.61	0.91	1.18	1.29± 0.27

As previously observed,[41] the results listed in **Table S3** show how RM1/MM method is giving lower values of ^{QC}KIE than those computed with M06-2X/MM. Finally, as observed in **Table S4**, quantum tunnelling effects additionally increase the computed quasi-classical KIEs by 1.29 ± 0.27 folds.

9 References

1. A. R. Nödling, K. Świderek, R. Castillo, J. W. Hall, A. Angelastro, L. C. Morrill, Y. Jin, Y.-H. Tsai, V. Moliner and L. Y. P. Luk, *Angew. Chem., Int. Ed.*, 2018, **57**, 12478-12482.
2. T. Sano, W. M. Pandori, X. Chen, L. C. Smith and R. C. Cantor, *J. Biol. Chem.*, 1995, **270**, 28204-28209.
3. C. E. Paul, E. Churakova, E. Maurits, M. Girhard, V. B. Urlacher and F. Hollmann, *Bioorganic & medicinal chemistry*, 2014, **22**, 5692-5696.
4. C. E. Paul, S. Gargiulo, D. J. Opperman, I. Lavandera, V. Gotor-Fernández, V. Gotor, A. Taglieber, I. W. C. E. Arends and F. Hollmann, *Organic letters.*, 2013, **15**, 180-183.
5. A. Geddes, C. E. Paul, S. Hay, F. Hollmann and N. S. Scrutton, *J. Am. Chem. Soc.*, 2016, **138**, 11089-11092.
6. A. Gangjee, Y. Qiu and R. L. Kisliuk, *J. Heterocyclic Chem.*, 2004, **41**, 941-946.
7. W. Ren, W. Chang, J. Dai, Y. Shi, J. Li and Y. Shi, *J. Am. Chem. Soc.*, 2016, **138**, 14864-14867.
8. P. Mahto, N. K. Rana, K. Shukla, B. G. Das, H. Joshi and V. K. Singh, *Org. Lett.*, 2019, **21**, 5962-5966.
9. A. B. Sanford, T. A. Thane, T. M. McGinnis, P.-P. Chen, X. Hong and E. R. Jarvo, *J. Am. Chem. Soc.*, 2020, **142**, 5017-5023.
10. D. T. Payne, Y. Zhao and J. S. Fossey, *Sci. Rep.*, 2017, **7**, 1720.
11. B. Qin, Xiao, X. Liu, J. Huang, Y. Wen and X. Feng, *J. Org. Chem.*, 2007, **72**, 9323-9328.
12. A. Geddes, C. E. Paul, S. Hay, F. Hollmann and N. S. Scrutton, *J. Am. Chem. Soc.*, 2016, **138**, 11089-11092.
13. T. Wymore, *ChemBioChem*, 2009, **10**, 2541-2541.
14. P. C. Weber, D. H. Ohlendorf, J. J. Wendoloski and F. R. Salemme, *Science*, 1989, **243**, 85.
15. W. L. Jorgensen, J. Chandrasekhar, J. D. Madura, R. W. Impey and M. L. Klein, *J. Chem. Phys.*, 1983, **79**, 926-935.
16. J. C. Phillips, R. Braun, W. Wang, J. Gumbart, E. Tajkhorshid, E. Villa, C. Chipot, R. D. Skeel, L. Kalé and K. Schulten, *J. Comput. Chem.*, 2005, **26**, 1781-1802.
17. Y. Duan, C. Wu, S. Chowdhury, M. C. Lee, G. Xiong, W. Zhang, R. Yang, P. Cieplak, R. Luo, T. Lee, J. Caldwell, J. Wang and P. Kollman, *J. Comput. Chem.*, 2003, **24**, 1999-2012.
18. J. Wang, R. M. Wolf, J. W. Caldwell, P. A. Kollman and D. A. Case, *J. Comput. Chem.*, 2004, **25**, 1157-1174.
19. J. Wang, W. Wang, P. A. Kollman and D. A. Case, *J. Mol. Graph. Model.*, 2006, **25**, 247-260.
20. S. E. Feller, Y. Zhang, R. W. Pastor and B. R. Brooks, *J. Chem. Phys.*, 1995, **103**, 4613-4621.
21. M. J. Field, M. Albe, C. Bret, F. Proust-De Martin and A. Thomas, *J. Comput. Chem.*, 2000, **21**, 1088-1100.
22. A. Krzemińska, P. Paneth, V. Moliner and K. Świderek, *J. Phys. Chem. B*, 2015, **119**, 917-927.
23. G. B. Rocha, R. O. Freire, A. M. Simas and J. J. P. Stewart, *J. Comput. Chem.*, 2006, **27**, 1101-1111.

24. M. J. Frisch, G. W. Trucks, H. B. Schlegel, G. E. Scuseria, M. A. Robb, J. R. Cheeseman, G. Scalmani, V. Barone, G. A. Petersson, H. Nakatsuji, X. Li, M. Caricato, A. Marenich, J. Bloino , B. G. Janesko , R. Gomperts , B. Mennucci , H. P. Hratchian , J. V. Ortiz , A. F. Izmaylov , J. L. Sonnenberg , D. Williams-Young , F. Ding , F. Lipparini, F. Egidi , J. Goings , B. Peng , A. Petrone , T. Henderson , D. Ranasinghe , V. G. Zakrzewski , J. Gao , N. Rega , G. Zheng , W. Liang , M. Hada , M. Ehara , K. Toyota , R. Fukuda , J. Hasegawa , M. Ishida , T. Nakajima , Y. Honda , O. Kitao , H. Nakai , T. Vreven, K. Throssell, J. A. Montgomery Jr. , J. E. Peralta , F. Ogliaro , M. Bearpark , J. J. Heyd , E. Brothers , K. N. Kudin , V. N. Staroverov , T. Keith , R. Kobayashi , J. Normand , K. Raghavachari , A. Rendell , J. C. Burant , S. S. Iyengar , J. Tomasi , M. Cossi , J. M. Millam , M. Klene , C. Adamo , R. Cammi , J. W. Ochterski , R. L. Martin , K. Morokuma , O. Farkas , J. B. Foresman and D. J. Fox, *Journal*, 2016.
25. G. M. Torrie and J. P. Valleau, *J. Comput. Phys.*, 1977, **23**, 187-199.
26. S. Kumar, J. M. Rosenberg, D. Bouzida, R. H. Swendsen and P. A. Kollman, *J. Comput. Chem.*, 1992, **13**, 1011-1021.
27. G. S. Grest and K. Kremer, *Phys. Rev. A*, 1986, **33**, 3628-3631.
28. Y. Zhao and D. G. Truhlar, *Theor. Chem. Acc.*, 2008, **120**, 215-241.
29. J. C. Keck, *Advances in Chemical Physics*, John Wiley & Sons, Inc., 2007.
30. S. Glasstone, K. J. Laidler and H. Eyring, *The Theory of Rate Processes: the Kinetics of Chemical Reactions, Viscosity, Diffusion and Electrochemical Phenomena.*, McGraw-Hill Book Company, inc, New York, 1941.
31. D. G. Truhlar, B. C. Garrett and S. J. Klippenstein, *J. Phys. Chem.*, 1996, **100**, 12771-12800.
32. C. Alhambra, J. Corchado, M. L. Sánchez, M. Garcia-Viloca, J. Gao and D. G. Truhlar, *J. Phys. Chem. B*, 2001, **105**, 11326-11340.
33. M. Garcia-Viloca, C. Alhambra, D. G. Truhlar and J. Gao, *J. Comput. Chem.*, 2003, **24**, 177-190.
34. J. Pu, J. Gao and D. G. Truhlar, *Chem. Rev.*, 2006, **106**, 3140-3169.
35. D. G. Truhlar, J. Gao, C. Alhambra, M. Garcia-Viloca, J. Corchado, M. L. Sánchez and J. Villà, *Acc. Chem. Res.*, 2002, **35**, 341-349.
36. D. G. Truhlar, J. Gao, M. Garcia-Viloca, C. Alhambra, J. Corchado, M. Luz Sanchez and T. D. Poulsen, *Int. J. Quantum Chem.*, 2004, **100**, 1136-1152.
37. M. Garcia-Viloca, D. G. Truhlar and J. Gao, *Biochemistry*, 2003, **42**, 13558-13575.
38. J. Pang, J. Pu, J. Gao, D. G. Truhlar and R. K. Allemann, *J. Am. Chem. Soc.*, 2006, **128**, 8015-8023.
39. K. Świderek and P. Paneth, *Chem. Rev.*, 2013, **113**, 7851-7879.
40. G. D. Ruggiero, S. J. Guy, S. Martí, V. Moliner and I. H. Williams, *J. Phys. Org. Chem.*, 2004, **17**, 592-601.
41. K. Świderek, K. Arafet, A. Kohen and V. Moliner, *J. Chem. Theory Comput.*, 2017, **13**, 1375-1388.
